# Cyclization in random graph modeling of acrylate copolymerization

**DOI:** 10.1039/d5sm01127b

**Published:** 2025-12-17

**Authors:** Tamika van 't Hoff, Teun Schilperoort, Ivan V. Kryven, Piet D. Iedema

**Affiliations:** a University of Amsterdam, Van’t Hoff Institute for Molecular Sciences Amsterdam 1098 XH the Netherlands p.d.iedema@uva.nl; b Mathematical Institute, Utrecht University Utrecht 3584 CD the Netherlands

## Abstract

Despite its many applications, three-dimensional radical polymerization remains poorly understood. A major challenge is the considerable kinetic slowdown caused by gelation—a liquid-to-solid phase transition that produces a network permeating the entire volume. This rapidly developing structure greatly obscures direct experimental observations of kinetic mechanisms during network formation. Although molecular dynamics (MD) simulations can qualitatively reproduce the gelation process, they are restricted to unrealistically short time scales. To address this limitation, particularly with respect to cycle formation, we propose coarse-grained modeling techniques based on random graphs (RG) and Monte Carlo (MC) simulations, and apply them to the polymerization of (multi)functional acrylates: *N*-butyl acrylate (NBA), 1,6-hexanediol diacrylate (HDDA), and trimethylolpropane triacrylate (TMPTA). This approach emphasizes the network of monomer units in the polymer rather than representing individual molecules in atomistic detail. In our models, cycles are represented as special types of vertices, depending on their size. The model demonstrates the impact of cycles, such as a delay in the gel point, which varies with cycle size. The number and size of cycles predicted by the coarse-grained models agree well with MD simulations, but they still fail to capture certain structural features, such as overlapping cycles. Typically, in the gel regime, RG and MC models predict structures with many connected cycles essentially in a tree-like pattern.

## Abstract 2

Despite its many applications, three-dimensional radical polymerization remains poorly understood.

## Graphical abstract

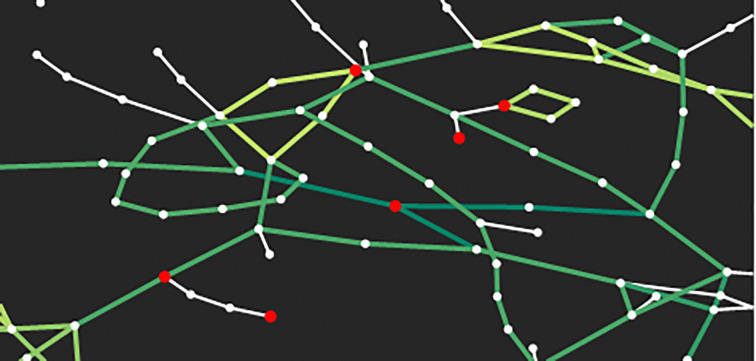

## 1 Introduction

Three-dimensional radical polymerization is a complex process, where multifunctional monomers react to form network structures under drastically changing conditions, namely a liquid-to-solid phase transition. These systems are both theoretically challenging and relevant in practice for a wide range of applications that include photo-polymerization of multifunctional acrylates for industrial printing and dentistry^1–4^ and autoxidative drying of linseed oil in oil painting – where unsaturated fatty acid esters act as the higher-functional monomer units.^5^ Mathematical modeling of 3D polymer networks had already been a topic of interest since the 1980s in the previous century, starting with the pioneering work by – among many others – Karel Dusek,^6,7^ which resulted in several new modeling approaches. In recent years, 3D-radical (co)polymerization has received increased interest, especially after its scope widened to include controlled radical polymerization.^8–14^ Apart from the useful applications, 3D-polymerization has become an intriguing topic of theoretical and experimental studies in view of its assumed inherent capability – either desired or unwanted – of spontaneously forming heterogeneous polymer networks, either in radical systems^15–23^ or in other, for example an organosilica network.^24^ This phenomenon is related to the complexity of the polymerization processes of multifunctional monomers that undergo a rapid liquid-to-solid phase transition, causing a dramatic decrease in reaction rate values. The fast and tremendously changing structure greatly obscures the direct observation of the kinetic mechanisms in 3D polymerization. This is in contrast to linear polymerization, where kinetic rates are more directly inferred using established characterization techniques like size exclusion chromatography, revealing the molar mass distribution. Knowing the polymer structure in linear polymerization is much less a prerequisite to uncovering kinetics than it is for 3D polymerization.

The formation of cycles in polymer networks has been a frequently discussed issue. We have argued in previous work that formation of small cycles (one or two monomer units) delays the gel point.^25^ Later on, we showed the occurrence of a hierarchy of clustered cycle structures with cell complexes with connected ‘holes’.^26^ An interesting theoretical approach, using Monte Carlo simulations, has recently been followed by Tobita.^27,28^ In this work cycle formation is an important issue, but – contrast to the present study – no explicit cyclization kinetics were addressed.

A further issue is the timing of the cycle formation. According to ref. 19–22 heterogeneous polymer networks are formed as clusters of microgels that are created earlier in the polymerization process by intensive and local cyclization. Hirokawa *et al.*^18^ found experimental evidence of such microgel formation in a system with a crosslinker. In contrast, another theory is that the process initially forms a homogeneous polymer network.^11,12^ Only at later stages does cyclization occur between reactive groups that are close together and are still accessible with their restriction mobility, eventually leading to heterogeneity. Despite the differences in these concepts, cyclization plays a crucial role in both assumptions. Questions involve the impact of cyclization on the gel point – do they indeed delay it? – and the timing – does cyclization happen right from the start, creating microgels, or only after substantial homogeneous gelation has already taken place?

**Fig. 1:**
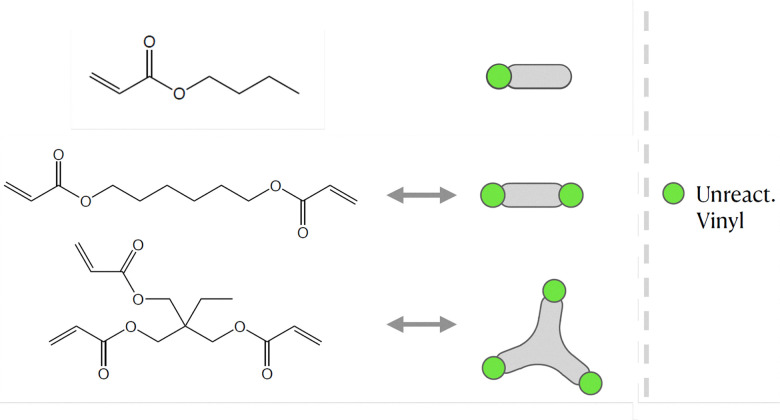
The molecular structure of the three unreacted monomer structures *N*-butyl acrylate (NBA), 1,6-hexanediol diacrylate (HDDA) and trimethylolpropane triacrylate (TMPTA) and their simplified notation. The unreacted vinyl groups that are essential for the polymerization processes are highlighted in the simplified notations on the right.

**Fig. 2:**
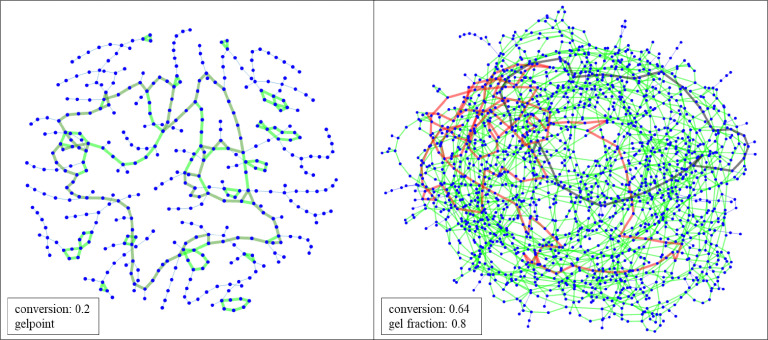
Poly-HDDA networks from molecular dynamics. Left: Largest component (401 monomer units) at gelpoint (maximum size second-largest component), vinyl conversion 0.2. 58 small (shortest path) cycles up to 26 units (light green). Many large cycles connecting small cycles, one shown in dark-green with length 95. Right: Largest component (1663 monomer units) well into gel regime, vinyl conversion 0.64, gel fraction 0.8. Around 800 small cycles up to 49 units (light green), largest one in black. Many large cycles connecting small cycles, one shown in red with length 121.

The objective of this paper is to shed light on cyclization in the radical copolymerization of mono-, di- and triacrylates: *N*-butyl acrylate (NBA), 1,6-hexanediol diacrylate (HDDA) and trimethylolpropane triacrylate (TMPTA), see Fig. 1, using data from atomistic modeling as input new macroscopic models, one based on random graphs (RG) and one on Monte Carlo (MC) sampling simulations. Molecular dynamics (MD) in a truly 3D atomistic modeling approach has recently been applied by our and other groups to describe the 3D polymerization of acrylates.^25,26,29–31^ In these works the formation of polymer molecules and giant components (gel) is simulated in a most realistic manner and provides a wealth of information about the connectivity structure of the networks produced and the kinetic rates at which this happens. Fig. 2 shows the result of an MD-simulation of 2000 HDDA-monomers (details in ref. 25) at the gelpoint (left) and well into the gel-regime (right). The figures clearly show an abundance of small cycles (defined by the shortest path, see also Section 2.2) and large cycles connecting small cycles. The drastic reduction in mobility has been quantified in terms of decreasing propagation and termination rate coefficients.^29^ In this paper, we will employ MD-data to quantify cyclization and derive the formation rates for cycles of different sizes.

Although successful and generating a wealth of data, MD is not a proper tool for engineering models in view of its computational expense. The macroscopic models we developed, RG and MC, are no longer based on a 3D description in space. Instead of molecules with atomic resolution, RG and MC use abstract ‘monomer’ units that specify only the key functional groups: unreacted vinyls, radicals and links with other units.^32^ Using population balance (PB) equations describing reaction rates, the concentration profiles of the units and the resulting degree distribution – describing the connectivity between the units – is calculated and serves as input to both RG and MC. The connectivity between real molecules observed in MD is directly comparable to the predicted connectivity between monomer units in RG and MC. In the present paper, we will employ MD-generated propagation, termination and cyclization rates as inputs for RG and MC. Polymer and network properties^32^ like size distribution, gel point and gel fraction computed from RG and MC using MD-generated rates can thus be directly related to the structural changes in MD, but at considerably lower computational cost.

To accommodate for cyclization in RG and MC, new features have to be added to the degree distribution used in previous research.^4,32^ We use a method inspired by Newman^33^ and Karrer^34^ and incorporate cycles as connected components bound *via* directed edges to distinguished cycle nodes. This requires extra kinetic information on cyclization to be included in the degree distribution. As this increases the complexity of the PB equations, we will apply the automatic reaction network generation (ARNG) approach successfully used before.^4,5,35–37^ Note that in ref. 4 we have employed kinetic rate coefficients, estimated by Abdi *et al.*^38^ from HDDA-conversion data measured by Fourier Transform Infrared spectroscopy. Abdi *et al.*^38^ do not provide kinetics of cyclization as a function of cycle size. In the present paper, focusing on cyclization, we will instead use fully MD-predicted kinetic coefficients for cyclization and for all other reactions.

The models developed in this study aim at predicting structural properties of acrylate networks that are not easily accessible for direct experimental validation. However, the kinetic data used have a firm basis in extensive experimental data (*e.g.* Abdi *et al.*^38^) Furthermore, the HDDA-structure predicted by MD provided good agreement with experimental data on glass transition temperature and Young's modulus.^25^ Finally, predicted polymer properties like gelpoint and gel fraction and size distribution can in principle to be checked with polymer characterization techniques like size-exclusion chromatography (SEC) or viscosimetry (DV).

This paper is structured as follows. First all the elements of our modeling approach are introduced and focused on the application: photo-polymerization of multifunctional acrylates. It is explained how cyclization rates are inferred from MD-data. The new method to accommodate cycles in RG and MC using distinguished in- and outgoing edges is described. Descriptions of the new RG-model and the MC sampling procedure are given. In the Results section we present the cyclization rate coefficients as a function of cycle size obtained from MD. As intermediate outcomes from ARNG and PB equations we discuss some time profiles of monomer species and degree fraction concentrations. Then polymer properties such as a bivariate size/cycle distribution, gelpoint and gel fraction are presented. The impact of cyclization as a delaying influence on the gelpoint is found. Finally, we compare the structures going from MD to those of the macroscopic model, from MC, noting a few interesting differences.

## 2 Modeling approach

**Fig. 3:**
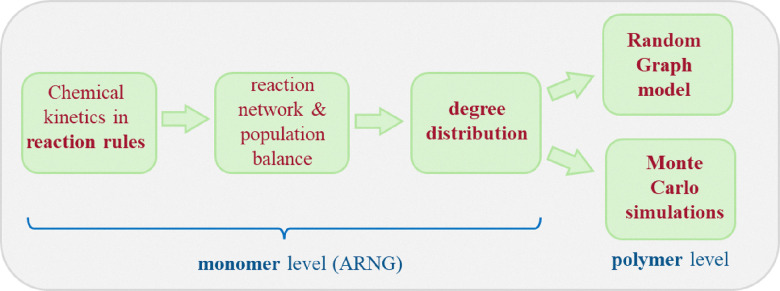
Overall modeling scheme. At the level of monomer units the automatic reaction network generator provides the reaction network, which automatically constructs the population balance equations. The degree distribution forms input for both random graph model and Monte Carlo simulations computing properties at polymer level.^5^

The 2-level overall modeling approach is depicted in Fig. 3. At the level of monomer units the Automatic Reaction Network Generator (ARNG) is responsible for creating the reaction network and automatic construction of the population balance (PB) equations (normally prepared manually). Solving the PB system provides the degree distribution, which describes connectivity: number and type of connections between monomer units. We will briefly call these connections linkages (avoiding the confusing ‘crosslinks’), synonymous to the graph-theoretical term edges.

At the second level polymer properties are computed by a random graph model and Monte Carlo simulations.

This section starts with a general description of the kinetic scheme of acrylate photopolymerization. This is followed by a discussion on the identification of cyclization rate coefficients from MD-data in relation to those for propagation and termination. Subsequently, the deterministic part of the modeling is presented. First, the new way of implementing cycles in random graphs is explained. Then a description follows of the way cycles have been implemented in the ARNG-algorithm that generates the PB-equations (monomer level – see Fig. 3). Next, at the polymer level, the cycle concept is introduced in the RG-model. This section concludes with a description of the stochastic modeling part: Monte Carlo sampling.

### 2.1 Free radical photo-polymerization of multifunctional acrylates

We adopt the kinetic scheme of free radical polymerization of multifunctional acrylates as introduced in earlier work^4,38^ with the usual steps: dissociation of initiator, initiation of radicals, radical propagation and termination – see Appendix A.1 and Fig. 14.

Since this paper is focused on cyclization, we highlight this mechanism here. Although cyclization reactions lead to identical chemical bonds as radical-vinyl propagation reactions, they are treated differently. First, regular propagation is second-order, depending on radical and vinyl group concentrations. Cyclization happens when a radical attacks a pending vinyl group on the same chain and is therefore first-order in the concentration of those radicals. (see also ref. 39–41). Also, cycles of varying lengths can be created, see ref. 26, and the rates may depend on size. One might expect that the impact of cycles on network topology will depend on the number of monomer units in the cycle. Formation of monocycles by reaction of a radical and a vinyl group on the same monomer prevents the creation of pending vinyl groups and hence branching. Detailed cyclization kinetics will now be derived from data available from Molecular Dynamics simulations.

### 2.2 Identifying rates of cyclization reactions from MD data

Torres-Knoop *et al.* proposed a method that infers chemical rate coefficients from MD simulations.^29^ The MD data contain a time series of data pertaining to the changing position of atoms and, from there, the formation of bonds in 3D space. This microscopic information is sufficient to estimate the macroscopic rate coefficients, if the stochastic nature of the date is accounted for. In ref. 29, the extraction of macroscopic reaction rates has been performed by an inverted kinetic Monte Carlo (kMC) method. In this method the time series of the MD data are converted into vinyl conversion series according to:1
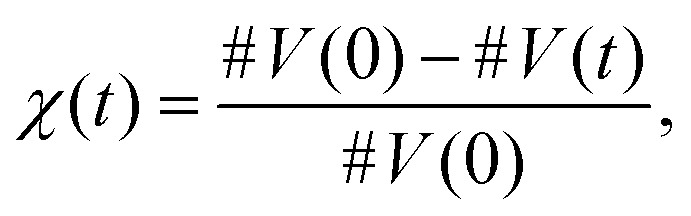
with #*V*(0) denoting the initial number of unreacted vinyl groups present in the system and #*V*(*t*) the number of unreacted vinyl groups left at time *t*.

The MD data used were obtained from Torres-Knoop *et al.*^26^ simulating a polymerizing HDDA system with 2000 monomers that react over a time period of 6.6 ns. We applied the inverted kMC approach to estimate from the atomistic MD data rate coefficients for all of the reactions relevant for this paper: propagation, termination and cyclization for sizes up to 54.

In order to calculate the rate of cyclization reactions, we need to count the number of cyclizations of different sizes occurring during the polymerization as simulated by MD. Cycle formation in MD is tracked and translated into an adjacency matrix containing the connectivity between the monomer units, the ‘nodes’. Then, a ‘shortest path’ method is employed: at each time step for all pairs of new bonds formed it is checked, whether at the previous time step they were already part of the same polymer molecule. If so, a cycle must have been formed, and the shortest path between them is the cycle length.

The reaction rate coefficient *k*_*c*_ for a specific cycle size *c* is estimated using the inverted kinetic Monte Carlo (kMC) method proposed for a 1st-order reaction by Torres-Knoop *et al.*:^29^2
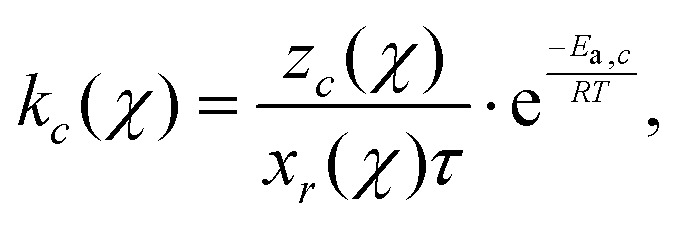
where *z*_*c*_(*χ*) denotes the number of cyclization reactions for cycle size *c* through conversion as observed in the MD simulations, *x*_*r*_(*χ*) is the number of radicals present at conversion *χ* and *τ* = 6 × 10^−12^ the discrete time interval. We assume that the activation energy of cyclization reactions equals the activation energy of propagation and has a value of *E*_a,*c*_ = *E*_a,p_ = 31.02 × 10^3^ J mol^−1^ and a temperature of 300 K. This is based on the similarity of changed energetic interactions on atomic level of propagation and cyclization reactions.

Solving the PB-model also requires values for the propagation and termination rate coefficients as a function of vinyl conversion. We have adopted the values for termination from ref. 29 and the values for propagation from the same source as well, but the latter with a correction for cyclization events. This is explained in detail in Appendix A.5.

### 2.3 Deterministic modeling: automated reaction network generation and random graph model

In this section, we will first discuss the manner in which cycles are introduced in the RG. Then we present the ARNG algorithm, especially focusing on reaction rules for cyclization. Finally, we describe how the new RG model with the capability for cycles is employed to compute polymer networks.

#### 2.3.1 Introduction of cycles in random graphs

**Fig. 4:**
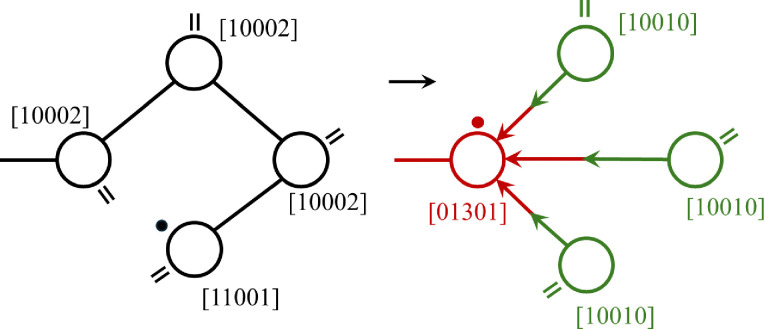
New cycle representation, formation of a cycle of size 4. The radical on the chain head attacks a vinyl group on the 4th unit. This creates in- and outgoing edges as expressed by the changes of the species vectors, [*v*, *r*, *i*, *o*, *u*]. The two reacting units change, but also the non-reacting intermediate units.

To accommodate cycles in the RG-model, we combine ideas from ref. 33 and 34 with the employment of ingoing and outgoing edges next to ‘undirected’ edges from ref. 32. The concept is shown for the formation of a cycle of four units in the case of a two-functional acrylate as shown in Fig. 4. As before, in ref. 4, for the RG-model we employ vector representations for the degree distribution, *u*(**k**), **k** = [*i*, *o*, *u*] labeled by the number of in *i*, out *o*, and undirected *u* edges, and the full species, **s** = [*v*, *r*, *i*, *o*, *u*], which is the degree distribution vector preceded by the number of vinyl (*v* and radical *r* groups). Note that according to this cycle representation both the ingoing and outgoing edges replace two undirected edges. An important feature of this cycle implementation is that within the cycle the attacked node is distinguished from the other nodes – they get different degrees. This reflects the different growth histories of the attacked and other units. The former – by its radical – can readily undergo further reactions with vinyl or radical, while the latter only at later stages will become activated by radical attack. In fact, all cycles of a size *n*_*c*_ have just one node with ingoing edges, generally having degree [*n*_*c*_ − 1, 0, *u*]. Note that this differs from the original representation by Newman *et al.*^33,34^ employing artificial cycle nodes, where nodes in a cycle are interchangeable and do not perform different roles. Thus, Fig. 4 shows the formation of 4-cycle after the reaction equation:[1, 1, 0, 0, 1] + 3[1, 0, 0, 0, 2] → 3[1, 0, 0, 1, 0] + [0, 1, 3, 0, 1].This expresses that head radical [1, 1, 0, 0, 1] (with one undirected edge) is reacting with a pending vinyl [1, 0, 0, 0, 2] in the same chain (with two undirected edges). The two intermediate units are not reacting themselves, but they change status becoming parts of the cycle.

The fact that attacked units get a different degree than other cycle units is important in view of the random connection procedure in the RG-model that employs the degree distribution input. It prevents the distinguished units to combine with itself. In discussing the results, we will revisit this issue. Note that the new cycle convention is applied to all cycles sizes except size one, created by reactions between vinyl and radical on the same unit. In our model only the disappearance of the vinyl group is accounted for.

Since the present work is the first attempt of implementing cycles in a RG-model framework, we have put restrictions on the possible cycle configurations. The main assumption is that each node can be part of only one cycle. A node with ingoing edges could become part of a second cycle and thus receive an outgoing edge as well, but we do not yet consider it at present. Likewise, nodes with one outgoing edge could become part of further cycles and get more outgoing edges, but we restrict the maximum of outgoing edges to one. Regarding Fig. 2 this assumption is certainly a significant simplification. One observes that cycles of different size share not only nodes, but even one or more edges. In principle, the node- and edge-sharing frequency can be inferred from the MD-data and employed in the RG-model. This is beyond the scope of the present paper, but an interesting topic for further investigations.

#### 2.3.2 Automatic reaction network generation

Traditionally, the construction of a kinetic model based on a reaction scheme like displayed in Fig. 14 happens in a manual way. In ref. 4 we have demonstrated that for mixes of multifunctional acrylates, it is worthwhile to apply an automated approach, automatic reaction network generation (ARNG). First, a bipartite reaction network with species and reaction node is constructed, based on reaction rules. From there the PB equations are automatically generated. In Appendix A.2 an illustration of the construction of reaction network and subsequent PB equations is given. Accounting for cycles turns out to lead to considerably more species and equations, as will be shown in the Results section.

The reaction rules including cyclization are presented in Tables 4 and 5 of Appendix A.3. The construction of the reaction network in general starts with just two initial species: the initiator I_2_ and unreacted monomer with two vinyl groups **n** = [2, 0, 0, 0, 0] (see Fig. 15 in Appendix A.3). Successively applying reaction rules to the species present generates new species until the number of species no longer increases. After the species list is completed, the model once more applies the reaction rules to the *n*_*s*_ different species to generate all the possible reactions, *n*_*r*_. Finally, all monomer species and reactions are collected in a bipartite graph that forms the reaction network. While in traditional modeling these numbers of species and reactions are predefined manually, here the size of the model is a result of the automated reaction network generation (ARNG) algorithm and the included model specifics, such as reaction types. The size and properties of the resulting model for multifunctional acrylates will be presented in the Results section.

The last part of the ARNG-procedure is generating PB equations. Previously, we were concerned with first- and second-order reactions that are easily described in PBE terms^4,5,35^ Cyclization is essentially first-order in the radical involved, but two complications arise. The first issue for a cyclization of size *c*_*s*_ to happen is, whether the radical will find a reactive unit at the same chain at a distance of *c*_*s*_ units – there may be none or several types. Secondly, as said before, intermediate units are present, whose change also should be accounted for in the PB equations. To limit complexity, we presently will assume that only 'head’ radicals will entail cyclization. This is a simplification indeed, as by inspecting the MD data in detail, one observes also chain radicals undergoing cyclization. The derivation of the governing equations will be presented in Appendix A.4. The result is:3
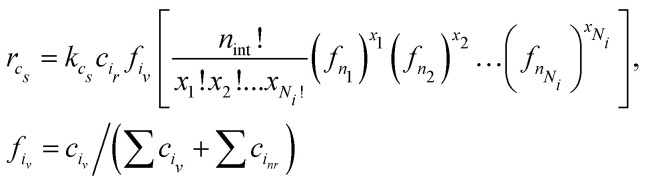
Here, *c*_*i*_*r*__ is the concentration of head radical unit *i*_*r*_ and *f*_*i*_*v*__ is the fraction of vinyl unit *i*_*v*_ of all chain units that the head radical can meet. The term in square brackets is a multinomial distribution denoting the probability, for a cycle of size *c*_*s*_ with *n*_int_ = *c*_*s*_ − 2 intermediate units, of finding a specific set of intermediate units, [*x*_1_, *x*_2_, …, *x*_*N*_*i*__]. *N*_*i*_ is the number of possible intermediate units, while the number of elements in the set is *n*_int_ so 
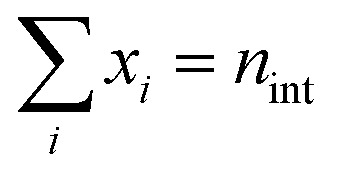
. Eqn (3) well demonstrates that for larger cycles the many possible combinations will lead to a significant number of PB equations.

#### 2.3.3 Computing polymer properties with the RG model

The equations employed to compute the global or polymer properties with the RG model are listed in Table 1. They have been derived in previous publications^4,37,42^ and will be briefly discussed here in their specific application to multifunctional acrylates with cyclization. We will apply this approach to bivariate size/cycle distributions.

**Table 1:** Equations of the RG model to compute the bivariate size-number of cycle nodes distribution *w*(*s*_1_, *s*_2_) and gel fraction *g*_f_

Name	Equations	
Degree distribution	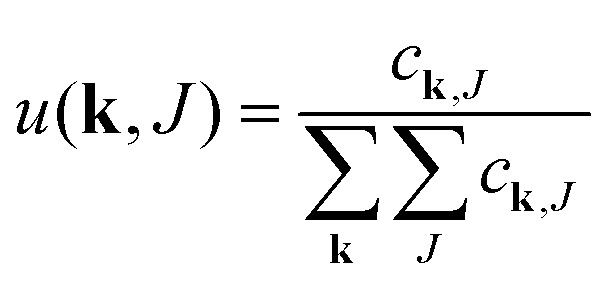	I
Excess deg. dist. edge type q	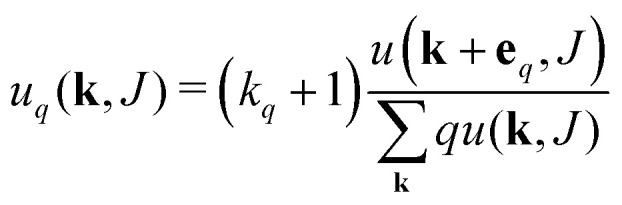	II
Generating functions	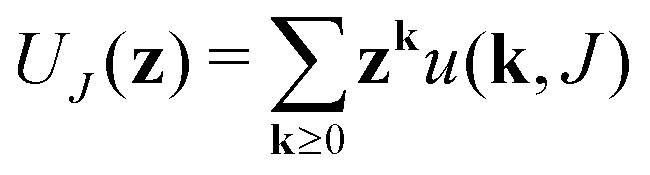	III
Of degree distribution	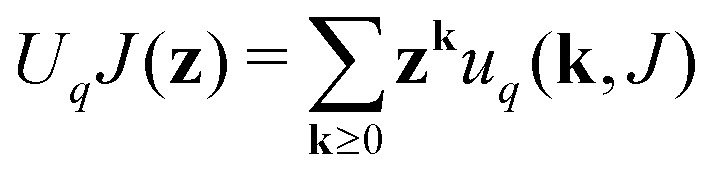	IV
Generating functions	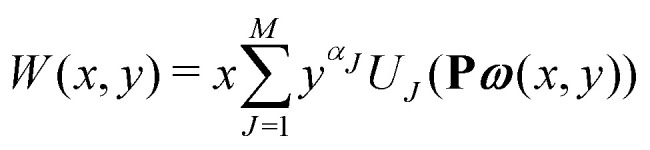	V
Of bivariate distributions	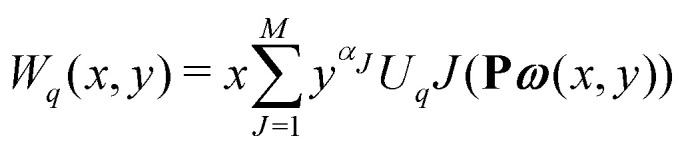	VI^a^
Gel fraction	*g*_f_ = *W*(1, 1)	VII
Size/mass- and size/cycles distributions	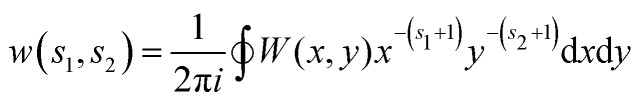	VIII

Permutation matrix	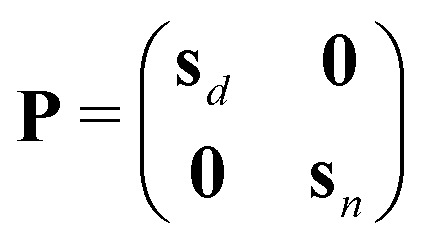	IX
Pairing rules	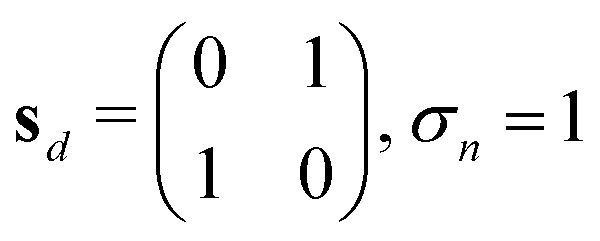	X

Definitions	*q* = 1, 2, 3; denotes edge-type	
	**k** = [*i*, *o*, *u*]	
	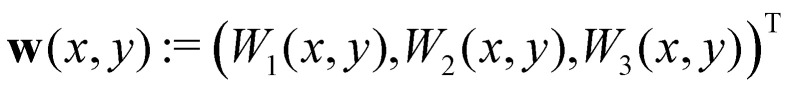	
	weighting vector: *a* = [1, 0]	

a Recursive equations.

The equations employed to obtain bivariate distributions are given in Table 1. Equations I and II express the degree distributions and the excess degree distributions of edge type *q* as obtained from *c*(**k**, *J*) (for each time point) by summations of all *j* = 1:*n*_*s*_ species concentrations over the numbers of vinyl *v*_*j*_ and radical groups *r*_*j*_. The generating functions of *u*(**k**, *J*) and *u*_*q*_(**k**, *J*) follow from Equations III and IV in a standard manner. When considering nodes without and with cycles the second dimension *J* has two possibilities, *J*_max_ = 2. This corresponds to the partitioning of the degree distribution in nodes without directed edges, *J* = 1, *i* = *o* = 0, and nodes with at least one directed edge *J* = 2, *i* + *o* > 0. Thus, *u*(**k**, *J*) is computed for all species *j* as:4
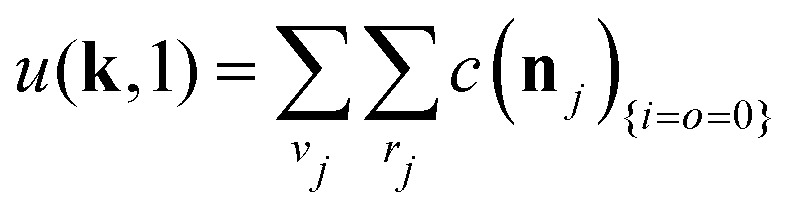
5
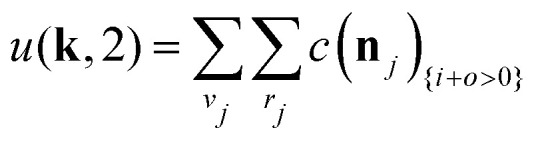
where we partition prior to summation. Eqn (4) and (5) create a distinction between nodes not within and within cycles, allowing to count the cycles in components of a given size. This is realized by applying weights *a*_*J*_ being used in Equations V and VI to assign a different weight to nodes within and not within cycles. Here we use *a* = [1, 0] implying that in the first dimension of the bivariate distribution only the nodes within cycles *s*_1_ = *c* are counted, while in the second all nodes are counted *s*_2_ = *s*. The overall monovariate solution for *s* is reproduced by summation over all *s*_1_ = *c*. Note that this approach is similar to van't Hoff *et al.*,^37^ where different molar masses were accounted for. This allowed to compute the mass distribution of copolymers by using different weight exponents for nodes with different monomer mass.

In Table 1, to establish the correct pairing of edges: *i* to *o* and *vice versa* and *u* to *u*, pairing rules are required, as shown in the second part of Table 1, Equations IX and X: a pairing matrix **P** and pairing rules *s*_*x*_. If **P**_*i*,*j*_ = 0, no bond is formed; otherwise, half-edge types *i* and *j* form a bond.

### 2.4 Stochastic modeling: Monte Carlo simulation

**Fig. 5:**
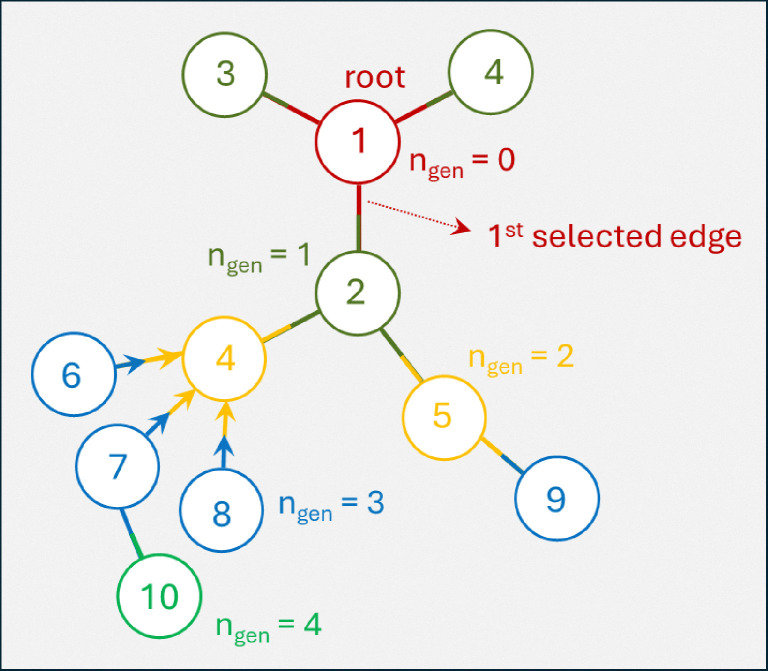
Illustration of Monte Carlo simulation procedure producing a molecule in 4 generations with 10 monomer units and one cycle of size 4. First sampling in generation 0 (red) is from the unbiased degree distribution, produces node 1 with 3 undirected edges, the ‘root’. In the 1st generation for each edge sampling takes place from the biased distribution. First selected edge generates node 2 with two further undirected edges (dark green, in total 3). The other two nodes connected to node 1 are node numbers 3 and 4, which have no further edges. Generation 2 (yellow): again sampling from biased distribution starting from node 2 generates nodes 4 and 5. Node 4 happens to be a cycle node with 3 ingoing edges, connected to nodes with outgoing edges, nrs 6, 7, 8. Node 5 turns out being connected with just one further undirected edge to node 9. Generation 3 (blue): nodes 6, 7, 9 have no further connection, but node 7 is connected once more to node 10. Generation 4: node *q*_0_ has no further connections.

Monte Carlo simulation is a useful tool for predicting polymer properties, while relatively simple to construct and to understand. There are various types of MC simulation models, here we employ a method introduced by Tobita (see *e.g.* ref. 43) and used by our group before (see *e.g.* ref. 44). The procedure we presently will use is mathematically equivalent to the RG-model and also produces the polymer properties like size distribution and gelpoint. The mathematical equivalence between RG and this type of MC becomes evident, when one considers the steps we take during the MC sampling procedure, demonstrated on an example in Fig. 5. Important to note that the input into the MC model is the same as the input of the RG-model: degree distributions *u*(**k**) and *u*_*q*_(**k**) (as described in Section 2.3.3).

The MC procedure starts, in generation 0, by random sampling of a node that may or may not be connected to other nodes and thus may be part of a polymer molecule, of which size and connectivity is not *a priori* known. The sampling takes place from the cumulative probability distribution (cdf) **F**, which is based on the degree distribution *u*(**k**), as will be shown below. The first selected node is called the root. The root may have (half-)edges through which it is connected further. In generation 1 for each of the edges the node type to which it is connected is sampled. Important is to note that connection rules apply: undirected edges, *u*, are connected to other such edges on the node to be connected, while given an outgoing edge, *o*, the connected node should have an ingoing edge *i*. Since the node in generation 1 is already connected, sampling has to take place from a different, biased cdf, **F**_*q*_. Clearly, the sampled node cannot have zero edges, while the probability of being sampled increases with (is biased towards) number of edges. This is consistent with the fact that the biased **F**_*q*_ is inferred from the excess degree distribution *u*_*q*_(**k**) – *i.e.* the multiplication with *k*_*q*_ + 1 in Equation II in Table 1. This sampling is repeated for all the edges in generation 1. If the nodes of generation 1 generate new edges, then a new generation of sampling has to take place. As in the 1st generation, and all subsequent generations, this happens from the biased cdf **F**_*q*_. The procedure stops when no further edges are found. This produces a polymer molecule, of which the numbers of nodes, eventually of a certain type, can be counted to obtain the size of the molecule as well as the number of cycles, or any other countable property. It is also possible that every generation generates new edges, which marks the formation of a gel – an infinite polymer molecule. In this case, to halt the computation, a maximum size of the molecule has to be set. Note finally, that the above MC procedure selects polymer molecules on a weight basis, *i.e.* larger molecules are sampled with higher probability.

Interestingly, the connectivity structure of each generated polymer molecule can be retrieved and stored as an adjacency matrix, the graph theoretical starting point for easy visualization and further graph properties, like shortest path between any two nodes. In fact, using graph tools MC sampling very conveniently allows to visualize the type of network obtained by the RG-model, which this model in itself cannot do. This is done in Section 3.6.

The aforementioned cumulative distribution functions **F** and **F**_*q*_ are derived as follows from degree and excess degree distributions *u*(**k**) and *u*_*q*_(**k**) defined in Equations I and II of Table 1. The number of elements in both vectors **F** and **F**_*q*_ is *n*_*u*_, the total number of different degrees **k** = [*i*, *o*, *u*]. The elements of the vectors follow as:6
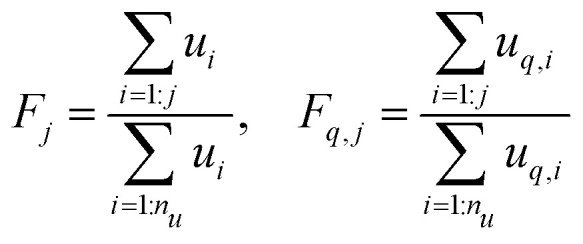


The sampling of a specific node *j* with degree [*i*_*j*_, *o*_*j*_, *u*_*j*_] from **F** proceeds by sampling random numbers:

7 *j* = max(rand() < **F**)

and likewise from the biased distribution **F**_*q*_.

## 3 Results

The combined automated reaction generation and random graph model is applied to various systems of polymerizing one-, two- and three-functional acrylates. In this section first the rates extracted from the MD data describing reacting HDDA are discussed for the various reaction types in the system. Then, we construct the reaction networks for the acrylates, where we assume that the rates are all equal those of HDDA. To describe the impact of cyclization on the polymer system, a series of computations are made accounting for an varying number of cycles.

### 3.1 Reaction rates of cyclization reactions

**Fig. 6:**
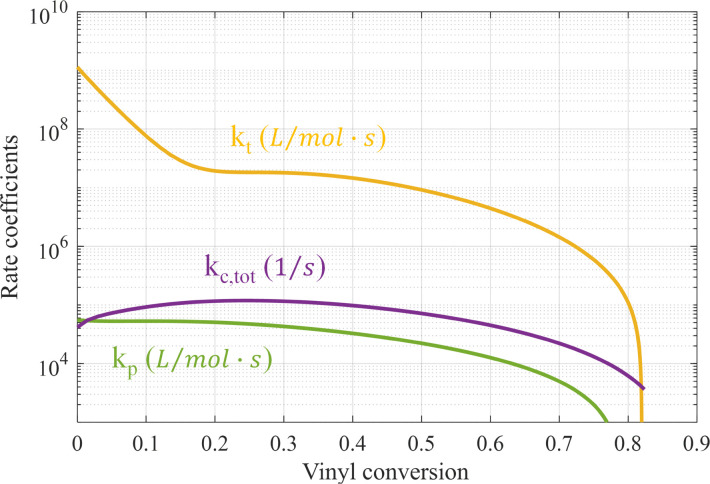
Rate coefficients of termination, propagation and the sum of all cyclization reactions using the inverted method proposed by ref. 29 as a function of conversion, see eqn (1).

To determine the cyclization and corrected propagation rate coefficients from the MD simulations, the inverted rate approach from Torres-Knoop *et al.*^25^ in combination with the shortest path method is employed, as explained in Section 2.2. For cyclization and corrected propagation, we fitted the reaction event frequencies *z*_p_ = *z*_*v*_ − *z*_*c*_ and *z*_*c*_ with 4th-order polynomials in vinyl conversion for cycle sizes up to 54. Both frequency data and polynomial coefficients are shown in Appendix A.5. The resulting propagation, termination and total cyclization (all cycle sizes taken together) rate coefficients as a function of vinyl conversion are shown in Fig. 6. Note that thus we realize consistency with respect to levels and conversion dependency of the kinetic parameters within the domain of the MD simulation. However, *k*_p_ and *k*_t_ values shown do not differ much from those in our earlier work^4,38^ and lead to conversion-time profiles identical to the experimentally observed profiles (see Fig. B1. in ref. 4).

**Fig. 7:**
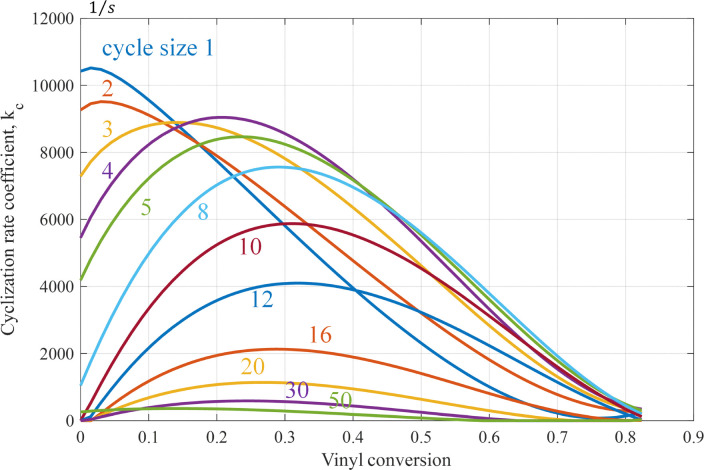
Cyclization rate coefficient as a function of vinyl conversion for various cycle sizes based on the fitted *z*_*c*_-data from ref. 29, see also Appendix A.5.

The cyclization rate coefficient as a function of cycle size and vinyl conversion based on the fitted *z*_*c*_-data is shown in Fig. 7. One observes that for smaller cycles, the rate coefficient just decreases with conversion. For a larger cycle, a maximum is seen at intermediate conversion. We may speculate about the reasons behind this behavior. Units close to the attacking radical might be reached easier than units further away. Also, due to the fast decrease of the termination rate, linear chains become longer, which may favor longer cycles. The fact that formation rates of larger cycles go through a maximum might be attributed to reactions with vinyls in smaller cycles already present, which might be closer and more exposed to the attacking radical than vinyls on linear chains. Any of these reasons will be explored in subsequent work, where we will study all the cyclization steps revealed by the MD simulation into more detail.

It is expected that there is competition between propagation and cyclization, in the sense that pending vinyl groups are either consumed by radical groups on other chains or by the terminal radical on the same chain. However, when comparing the number of propagation and cyclization reaction events (see Fig. 17), it is clear that the number of cyclization events is more than two orders of magnitude lower than the number of propagations. Hence, the required correction on the propagation rate is relatively small. On the other hand, since cyclization is a first-order reaction, the resulting cyclization coefficients still attain considerable values, as can be observed from Fig. 6 and 7.

### 3.2 Species and reactions generated by ARNG

Using the vector representation describing monomers and the reaction rules from Tables 4 and 5, we end up with reaction networks for polymerizing mixtures of NBA, HDDA and TMPTA. For HDDA assuming cycle sizes up to 4, the ARNG produces a reaction network that consists of 943 reaction nodes and 46 monomer species that are listed in Table 2. By collecting all species with the same degree **k** = [*i*, *o*, *u*] (or summation over *v* and *r*) the degree distribution as defined in Section 2.3.1 the degree distribution is found to have 17 different degrees. Some features of the species list deserve closer attention. Since it was assumed that a given monomer can only partake in one cycle, the maximum number of out-edges, *o* of a monomer species equals 1. Further note that even though the initial monomer used in the reaction network is the unreacted diacrylate (*M* = 3, 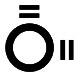), unbound monoacrylate is by default part of the network (*M* = 11, 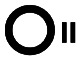) as it can be formed from disproportionation termination. Likewise, biacrylate can be formed when triacrylate (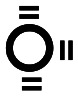) is the sole starting species. In the case of HDDA the maximum degree is 4 (6 for TMPTA), which is the monomer unit *M* = 21, 
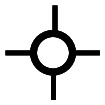
, [0, 0, 0, 0, 4]. Several distinguished cycle nodes are generated: with outgoing edges *M* = 23, 26, 27, 32, 38, 42 and 46, for instance those with two normal edges and one outgoing edge 
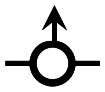
, [0, 0, 0, 1, 2] and with one, two and three ingoing edges: 
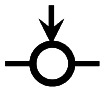
 – [0, 0, 1, 0, 2], 
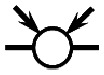
 – [0, 0, 2, 0, 2] and 
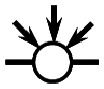
 – [0, 0, 3, 0, 2], respectively. Like 
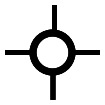
 these cycle nodes have also the maximum total degree of 4.

**Table 2:** List of 46 monomer species generated by reaction rules for HDDA with formation of cycles up to size 4. The dummy particle is used as placeholder in the reaction network generation and initially, only unreacted initiator (*M* = 2) and unreacted HDDA (*M* = 3) are present in the system. Collecting all species with the same degree **k** = [*i*, *o*, *u*] yields the degree distribution with 17 different degrees

*M*	*v*	*r*	*i*	*o*	*u*
1	Dummy				
2	Unreacted initiator: I_2_				
3	2	0	0	0	0
4	Initiator radical: I˙				
5	1	1	0	0	0
6	0	2	0	0	0
7	0	1	0	0	0
8	1	0	0	0	1
9	1	1	0	0	1
10	0	2	0	0	1
11	1	0	0	0	0
12	0	1	0	0	1
13	0	0	0	0	0
14	0	0	0	0	1
15	0	1	0	0	2
16	0	2	0	0	2
17	1	0	0	0	2
18	0	0	0	0	2
19	0	0	0	0	3
20	0	1	0	0	3
21	0	0	0	0	4
22	0	1	1	0	0
23	1	0	0	1	0
24	0	1	2	0	0
25	0	1	3	0	0
26	0	1	0	1	0
27	0	0	0	1	0
28	0	2	1	0	0
29	0	2	2	0	0
30	0	2	3	0	0
31	0	0	1	0	0
32	0	0	1	0	1
33	0	1	0	1	1
34	0	0	2	0	0
35	0	0	2	0	1
36	0	0	3	0	0
37	0	0	3	0	1
38	0	0	0	1	1
39	0	1	1	0	1
40	0	1	2	0	1
41	0	1	3	0	1
42	0	0	0	1	2
43	0	0	1	0	2
44	0	0	2	0	2
45	0	0	3	0	2
46	0	2	0	1	0

When applying ARNG to TMPTA assuming cycle sizes up to eight, one observes a clear illustration of the combinatorial explosion due to the many combinations possible of intermediate units in the larger cycles. This TMPTA reaction network encompasses 204 monomer species and 21 335 585 reaction nodes.

### 3.3 Time profiles of monomer concentration and degree distribution

Using the generated reaction network describing the polymerization of mono-, di- and triacrylates and the kinetic rates for propagation, termination cyclization as explained in Section 2.2 (based on MD-data), we construct population balances that describe the consumption and production of all monomer species through time. Thus, the time profiles of the numerous monomer species become available. Note that as in ref. 4 we choose initiator starting concentration in accordance to realistic photocuring conditions.^38^ We highlight and explain a few species profiles obtained for the case of a terpolymerization of NBA, HDDA and TMPTA with varying assumptions concerning cycle size. Although cyclization rate data are available from MD up till cycles of size 50, the full implementation would lead to excessive size of the equation system, as only 8 cycles for triacrylates already requires more than 20 million PB equations. We simplify by assuming the total cyclization rate attributed in proportion to sizes from 1 till 22 for HDDA and from 1 till 5 for systems with TMPTA. The impact of this simplification will be studied in this section. One important issue is the sensitivity of global polymer properties like the gelpoint to the maximum cycle size.

**Fig. 8:**
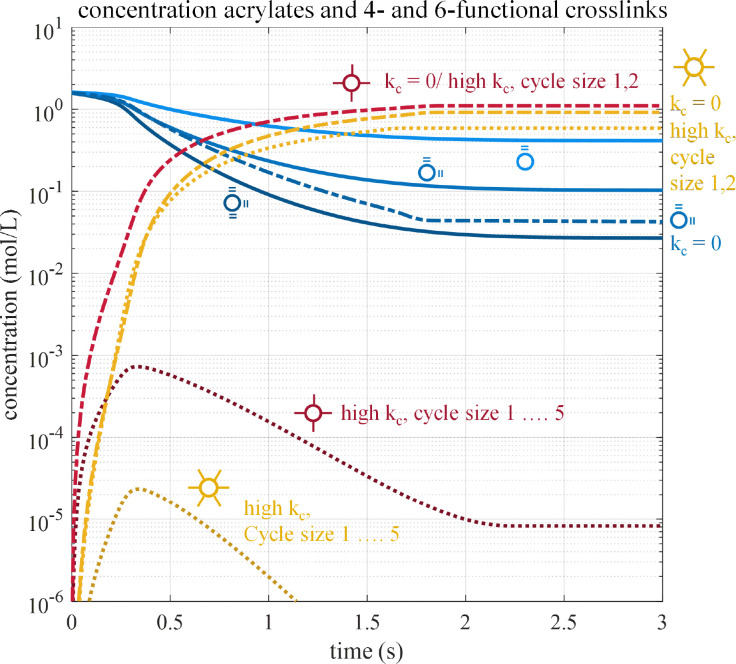
Time profiles of NBA, HDDA and TMPTA concentrations and concentrations of 4- and 6-functional linkages. In blue are acrylate concentrations assuming cyclization rates according to MD-data revealing faster consumption for higher-functional acrylates. In absence of cyclization (dark blue, only shown for HDDA) acrylate conversions are higher. In red and yellow are concentrations of 4- (
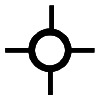
) and 6-functional (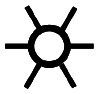) linkages. When cyclization is absent or produces only small cycles 
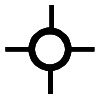
 and 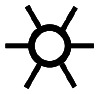 practically coincide. For larger cycles (up to size 5) 4- and 6-functional links attain much lower concentrations.

A few trends in the species concentrations and the degree fractions as functions of time or vinyl conversion are present, see Fig. 8 and 18 in Appendix A.6. Initially, only unreacted acrylates exist, being consumed as the polymerization goes on, being replaced by connected units. In 8 in varying shades of blue are the acrylate concentrations (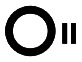, 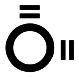 and 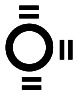) assuming cyclization rates according to MD-data, clearly showing that higher-functional acrylates are consumed more rapidly. Concerning cyclization, the total rate has been varied between zero, one and five times the MD-rate, while the maximum numbers of cycles have been varied (at constant total rate) between zero and five. In absence of cyclization (dark blue, only shown for HDDA) acrylate conversion is higher. In view of our assumption that the sum of propagation and cyclization events is constant (see Section 2.2), one might expect that the lower vinyl consumption by reduced propagation is compensated by increased vinyl consumption *via* cyclization. However, we have also limited cyclization to head radicals only (see Section 2.3.2), which are seen to be depleting causing vinyl consumption by cyclization to stop. Further trends, like those of 4- and 6-functional linkages and some cycle nodes are discussed in (Appendix A.6, Fig. 18). It is observed that linkage concentrations ultimately reach the level of initial monomer concentrations. Also larger cycles turn out to considerably suppress 4- and 6-functional linkage levels.

The degree distribution **k** = [*i*, *o*, *u*] is inferred from the species concentrations by summation over all numbers of vinyls *v* and radicals *r*. Fig. 19 in Appendix A.6 displays part of this distribution *versus* vinyl conversion for the case of HDDA polymerization with cycle sizes 1–8 forming according to MD-rates (total rate equal to total rate of all cycles observed in MD). Under these conditions 70 species are active in 16 807 reactions, yielding 29 different degrees. Fractions of degree 2 (linear chain segments) are relatively high at low conversion but at high conversion levels of 4-and 2-functional nodes are equal, indicative of a highly dense network. At high conversion the fraction of [0, 1, 2] nodes – with total linkage functionality 4 – considerably exceeds the level of the non-cycle nodes [0, 0, 4]. Hence, the reduced connectivity by fewer [0, 0, 4] nodes is compensated by the 4-functional cycle nodes [0, 1, 2].

### 3.4 Bivariate distribution of size and numbers of cycles per polymer molecule

**Fig. 9:**
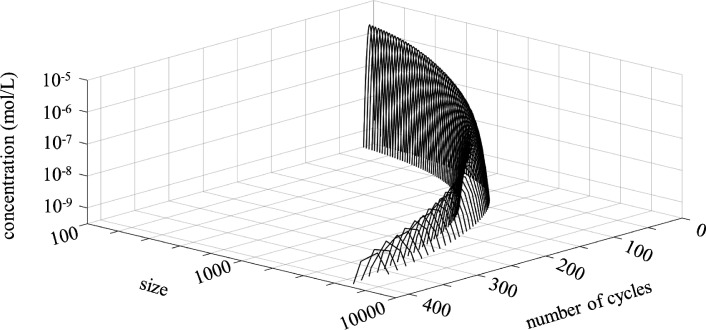
2-Dimensional distribution of size and number of cycles for poly-HDDA just before the gelpoint (vinyl conversion 0.0035) assuming maximum cycle size 7.

The bivariate component size-number of cycles distribution has been computed using the equations in Table 1 and kinetic rates based on MD-data (Section 2.2). An example is shown for poly-HDDA just before the gelpoint assuming maximum cycle length of 7 units in Fig. 9. Polymer molecules of around 5000 units possess between 350 and 400 cycles of varying sizes.

### 3.5 Influence of cycles on the gel point

In the Introduction, we have discussed the impact of cycles on gel formation rate and the expected delay of the gel point of a polymer, based on several literature ref. 11, 12, 18–22, 25 and 26. The questions we want to answer here are: does the RG model reveal a significant impact of cycle formation on gelation and does it indeed predict a delay of the gel point? Note that gel fraction here is defined as the weight fraction of gel compared to total weight of unreacted monomer and polymer.

Fig. 11 shows gel fraction *versus* vinyl conversion under varying assumptions concerning cyclization. One observes that cyclization indeed does delay the gelpoint, but only slightly. For pure HDDA (top) the predicted gelpoint is at vinyl conversion 0.0035, the curve in blue, corresponding to HDDA monomer conversion of around 1% (see also Table 3). For cyclization rates from MD the gelpoint shifts to conversion 0.004 (yellow curves). The maximum cycle size has been varied up to 22 units per cycle, while keeping the total cyclization size constant. Thus, for maximum cycle size of one the total cyclization rate as observed from MD is ascribed to cyclization of size 1. For a maximum of 22 all rates from 1 until 22 are taken in proportion to the values observed in MD (see Fig. 7), but adding to the same total rate.

**Table 3:** Gel fractions from random graph model (RG) and Monte Carlo simulations (MC) for HDDA-polymerization. Almost perfect agreement between RG and MC

Vinyl conversion	Fraction polymer^a^	Gel fraction (total^b^)		Gel fraction (polymer^c^)	
		RG	MC	RG	MC
4.16 × 10^−3^	7.59 × 10^−3^	4.29 × 10^−4^	4.42 × 10^−4^	0.0565	0.0582
4.43 × 10^−3^	8.08 × 10^−3^	1.09 × 10^−3^	1.12 × 10^−3^	0.135	0.138
4.97 × 10^−3^	9.06 × 10^−3^	2.40 × 10^−3^	2.42 × 10^−3^	0.265	0.267
6.63 × 10^−3^	1.21 × 10^−2^	6.22 × 10^−3^	6.25 × 10^−3^	0.516	0.518
9.67 × 10^−3^	1.75 × 10^−2^	1.28 × 10^−2^	1.27 × 10^−2^	0.728	0.725
2.50 × 10^−2^	4.46 × 10^−2^	4.23 × 10^−2^	4.23 × 10^−2^	0.948	0.947
7.17 × 10^−2^	0.123	0.123	0.123	0.995	0.993

a Equal to (1 – HDDA monomer conversion) ≈ (1 − *u*([0, 0, 0])).

b Weight fraction gel based on sum of moles of monomer bound in polymer and moles of unreacted monomer.

c Weight fraction gel based on total amount of polymer.

To test the sensitivity of the gelpoint for the intensity of cyclization, we varied the rate around the values found from MD. For total cyclization rate from MD and a factor 5 lower (yellow and green) hardly any impact of cycle size is observed. For a factor 5 higher rate the gelpoint delay is reduced as maximum cycle size is increased (red). Hence, the maximum effect is seen for mono-cyclization (self-loops). The fact that larger cycle sizes reduce the delay somewhat is explained that the decrease of 4-functional linkages is partly compensated by cycles that effectively form higher-functional semi-linkages. The effect stops at cycles of size 10, since at these low conversions larger cyclizations do not yet happen (see Fig. 7).

The bottom plots of Fig. 20 show the situation for terpolymerization of NBA/HDDA/TMPTA (left), which are not much different from those for pure HDDA, and for mainly NBA with only 5% HDDA (right). In latter case the gelpoint happens at much higher conversion and also the delays are more significant. We remark here that for pure high-functional acrylates the gelpoint occurs at very low monomer conversion. In such a situation there is no question of a ‘liquid–solid’ transition as after the gelpoint the system is best described as a network fluid. However, in the case of low multifunctional acrylate content, the gel transition happens at much higher polymer concentration and might therefore be better accessible to experimental detection.

**Fig. 10:**
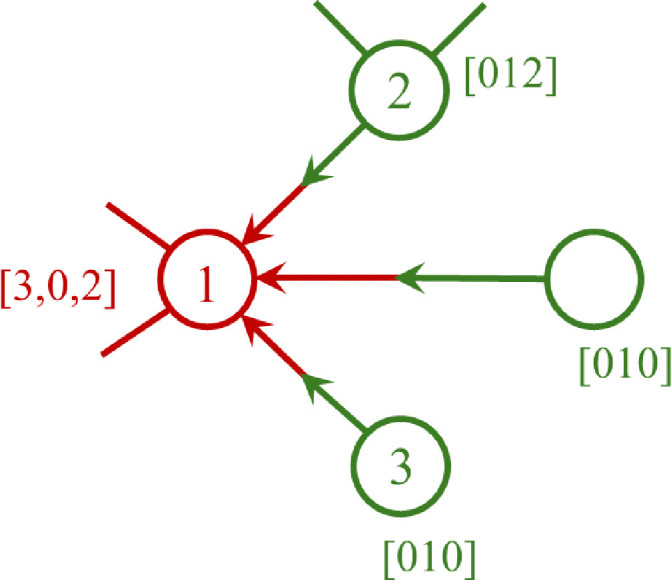
Cycle node combination 1 and 2 forms effectively a 4-functional linkage. Combination 1 and 3 forms 2-functional link. At gelpoint combination 1–3 dominates, at high conversion combination 1–2.

The gelpoint delay can be better understood by studying the behavior of the degree fractions around the gelpoint. In Section 2.3.1 the idea of distinguishing the attacked cycle node (characterized by ingoing edges) from all the other nodes (outgoing edges) in the cycle was introduced. Appendix A.7 and Fig. 20 further illustrate the impact of this distinction in detail. The reasoning is briefly as follows. It should be realized that gelation in HDDA is primarily caused by 4-functional linkages 
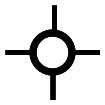
, which are reduced by cyclization. However, combinations of two nodes in a cycle may also effectively lead to 4-functional linkages, for instance the combination of nodes 1 and 2 in Fig. 10. At low conversion the level of nodes with ingoing edges like node 1 is relatively high, but the node 2 level is still very low and hence only slightly compensates the reduction of normal linkages 
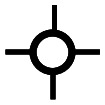
. Nodes with outgoing edges that do happen to be present at high concentrations are of type 3, but these do not have undirected edges and therefore do not form effectively 4-functional linkages in combination with node type 1.

The absence of effective cycle node combinations forming linkages also nicely illustrates why we need the distinction between ingoing and outgoing cycle nodes in the first place. If radical-attacked nodes in a cycle would not be distinguished from the other cycle nodes (by exclusively getting ingoing edges) combinations of two or more attacked nodes, *e.g.* of type 1 would become allowed. As said, the levels of such nodes are high, which would make their combination very likely. In a variant of our RG-model without the distinction between attacked and non-attacked units we observed the gelpoint indeed to be situated at extremely low vinyl conversion – as low as 10^−6^.

When discussing impact of cyclization on gelpoint, one might want to directly compare the RG-prediction to the MD-result. After all, the cyclization kinetics in RG have been extracted from data in an MD simulation that also describes a transition into the gel regime, see ref. 25. However, as has been put forward in this publication, the MD simulation had to be conducted at an order of magnitude higher radical concentration in order to obtain statistically representative results. It was observed and explained that this leads to a much higher gelpoint conversion. This complicates the direct comparison of gelpoint prediction by MD to that by RG, for which we choose to employ realistic radical levels.

**Fig. 11:**
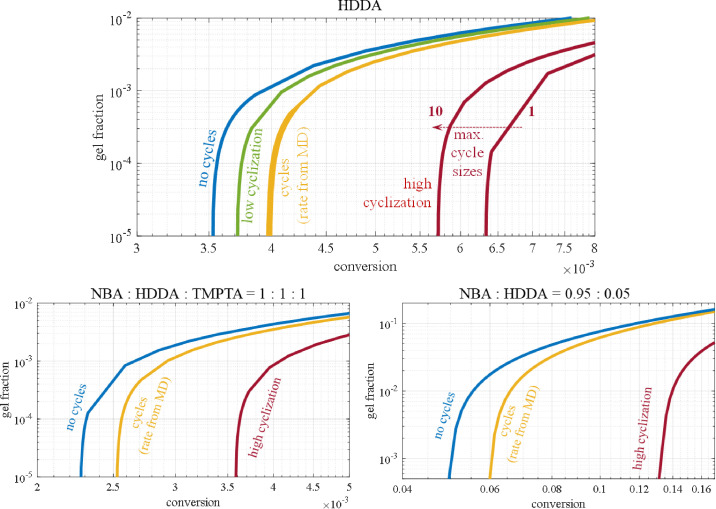
Impact of cycles of varying sizes and sensitivity to cyclization intensity on gelpoint vinyl conversion. Gel fraction is weight fraction gel based on total amount of monomers. Top. Cyclization in pure HDDA at rates obtained from molecular dynamics (yellow) lead to a small delay of the gelpoint: instead of 0.0035 (blue) 0.004. The delay is practically independent of the maximum cycle size, like it is for a factor 5 lower cyclization rates (green). For a factor 5 higher rates the delay is increased to conversions around 0.006, while larger cycle size lead to slightly smaller delays. Bottom left. Gel point delay for NBA/HDDA/TMPTA terpolymerization at MD-rates and a factor 5 higher at slightly lower conversions than pure HDDA. Bottom right. Gelpoint of mixtures of mostly NBA and 5% HDDA without cyclization (blue) at conversion 0.05 shifting to 0.06 for cyclization rates from MD and to 0.12 for higher rates.

### 3.6 Results of Monte Carlo simulations

**Fig. 12:**
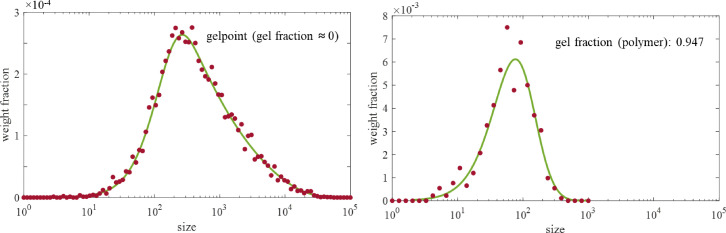
Size distribution of poly-HDDA, green curves: RG-model, red dots: Monte Carlo simulation. RG and MC with the same degree distribution input. Left. Distribution at gelpoint, vinyl conversion 0.0035. Right. Distribution well into gel regime, vinyl conversion 0.025, gel fraction of polymer: 0947 (see also Table 3).

Monte Carlo simulations have been executed to obtain size distributions of poly-HDDA based on populations of 10 000 or 100 000 generated polymer molecules for a case where a maximum of cycle size 7 is assumed. Fig. 12 on left hand side shows the distribution at the gelpoint, at vinyl conversion 0.0035 – same conditions as in Fig. 9 showing the 2-dimensional size/cycle distribution. The RG-curve represents the summation over all numbers of cycles per molecule of the 2-dimensional distribution. Apart from a certain scatter, the MC-predicted size distribution (red dots) coincides with the one-dimensional size distribution computed with the RG-model (green). Note that in RG and MC we employed the same degree distribution as input. At right hand side in the Fig. 12 the RG- and MC-distributions are shown for higher vinyl conversion, 0.025, well into the gel regime. The gel fraction based on the total amount of monomers bound in polymer is around 95%. This implies that the very narrow distribution in the figure represents only 5% of the polymer present. The agreement between RG and MC is clear, despite the scatter in the MC data. Note also that the size distributions could be validated with size-exclusion chromatography (SEC) – in the gel regime after separating out the gel.

We have also compared the values of the gel fraction as predicted by RG and MC, the results are in Table 3. Gel fraction have been expressed both as weight fractions gel referring to the total amount of monomers bound in polymer and free monomer (as above) and to the amount of polymer only. Latter quantity is directly available from the generation of a number of polymer molecules in MC: it is the fraction of the molecules growing to infinity (and stopped at a maximum size). The table shows that there is almost perfect agreement between RG and MC in a range between 5 and 100 weight% of the polymer being gel. Below 5% and nearer the gelpoint distributions become very broad, requiring excessive time for the MC simulation to generate a representative set of polymer molecules. At 5% the maximum size for finite molecules was found to be 10^6^.

**Fig. 13:**
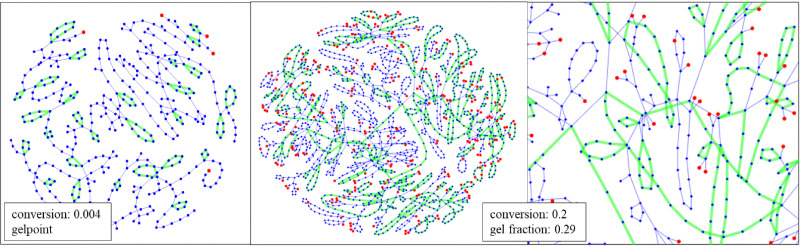
Poly-HDDA network fragments from Monte Carlo simulation, sizes comparable to MD networks in Fig. 2. Left. Part of infinite network at gelpoint, vinyl conversion 0.004. Displayed are 400 nodes, ed dots mark nodes, where the network is further extended but hidden from view. Cycles up to size 9 marked in green. Middle and right. Parts of network well into gel regime at gel fraction 0.29 (based on total monomer units) and vinyl conversion 0.2. 1800 nodes on display with red dots marking further connection. In green are adjacent cycles up to size 22 all connected by not more than one edge as shown in right plot. In blue are isolated cycles.

**Fig. 14:**
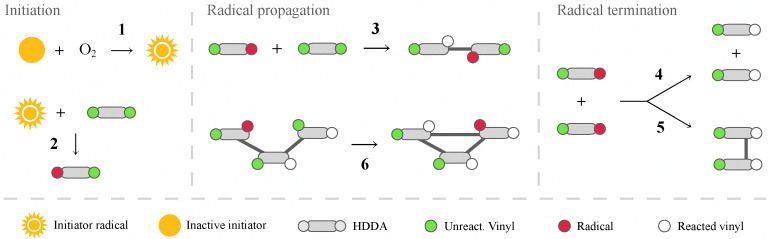
The free radical photo-polymerization of 1,6-hexanediol diacrylate (HDDA). The same representation of HDDA is used as in Fig. 1. The polymerization of HDDA starts with: (1) photo-initiation: initiator dissociation yields initiator radicals under the influence of ultraviolet light; (2) initiation by reaction between monomer and initiator radicals. (3) Propagation reactions. (4) Radical termination disproportionation. (5) Radical termination by recombination. (6) Cyclization reactions.

**Fig. 15:**
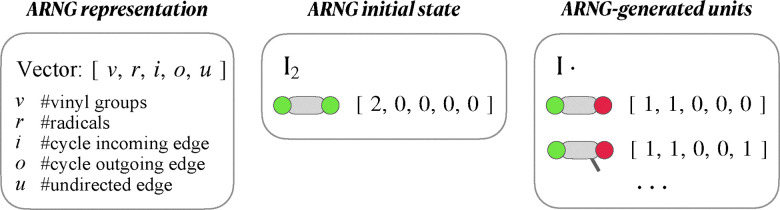
The 5-digit vector representation of monomer species used in the automated reaction network generation (ARNG). The vector includes the number of vinyl groups *v*, radicals *r*, in-coming edges (linkages) *i*, out-going edges *o* and undirected edges *u* a monomer species contains. Red dots: radical; green dots: vinyl group; white dots: reacted vinyl.

**Fig. 17:**
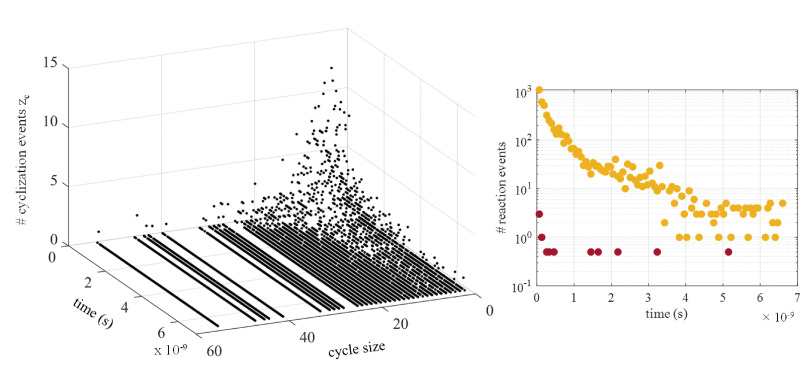
LHS: Cyclization events *z*_*c*_ as directly obtained from MD-simulations per cycle size. RHS: Propagation and termination reaction events from MD. Cyclization events are 2 orders of magnitude less frequent than propagation reactions.

**Fig. 18:**
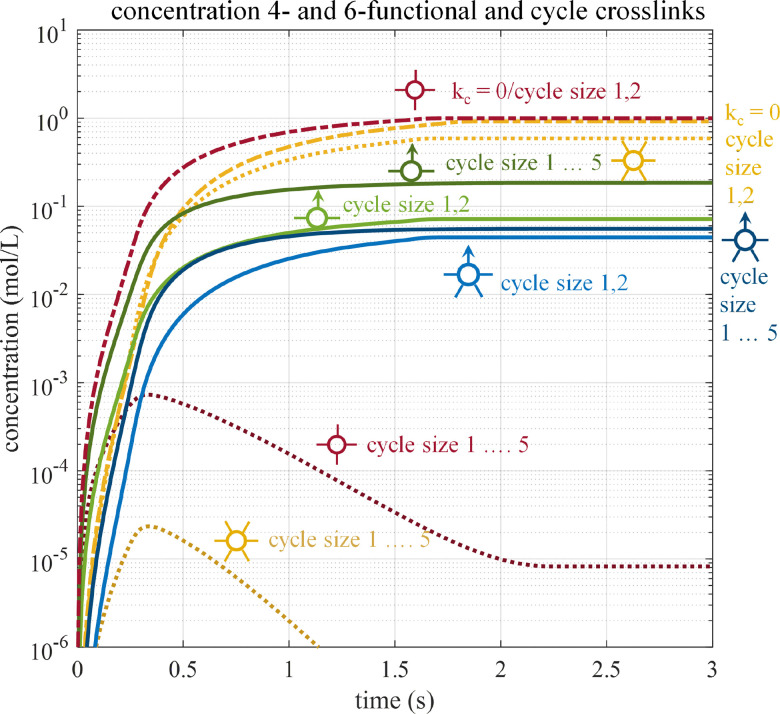
Comparison of 4- and 6-functional linkages to links inside cycles 
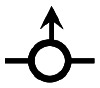
 in green (from HDDA) and 
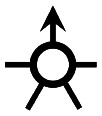
 in blue (from TMPTA). Larger cycle sizes lead to more cycle nodes.

**Fig. 19:**
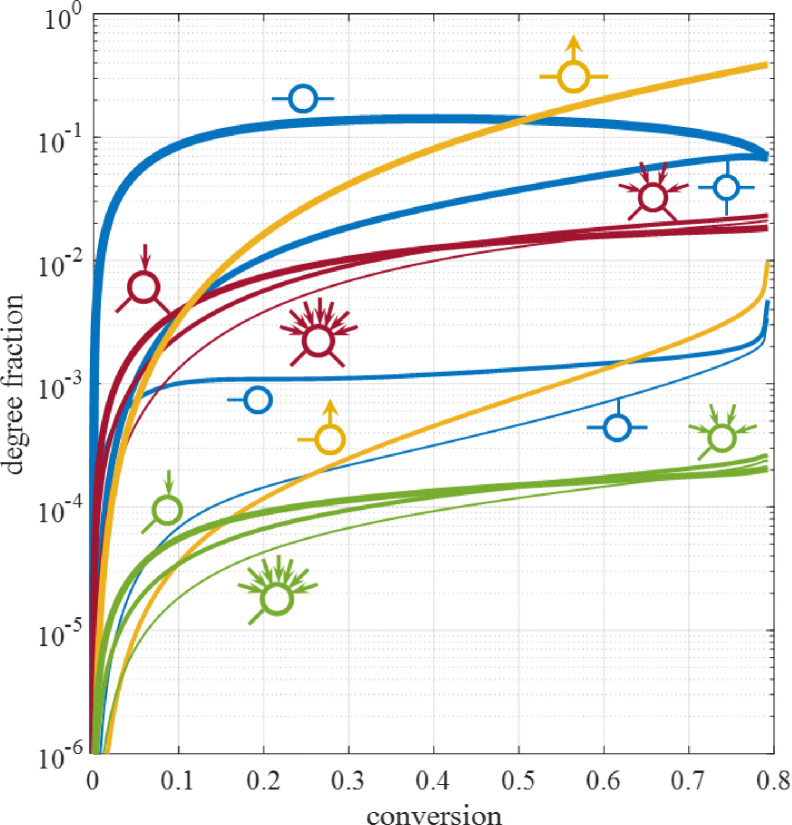
Degree distribution vs conversion for HDDA polymerization with cycle sizes 1–8 forming according to MD-rates. In blue with increasing numbers of normal edges, degrees *k* = [*i*, *o*, *u*] = [0, 0, 1], [0, 0, 2], [0, 0, 3], [0, 0, 4]. Those of degree and 2 (linear chain segments) are most abundant at low conversion but at high conversion levels of 4-and 2-functional nodes are equal. In yellow cycle nodes with an outgoing edge, at higher level with two normal edges (fat, degree [0, 1, 2]) than with one normal edge (thin, degree [0, 1, 1]). Cycle nodes with two normal edges and from one to 7 incoming edges: between 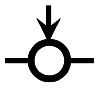 (degree [1, 0, 2]) and 
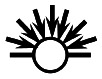
 (degree [7, 0, 2]) feature in red and those with one normal edge in green (degrees [1, 0, 1] and [7, 0, 1]). Since cyclization rate decreases with size, fewer nodes with a larger number of incoming edges are present.

**Fig. 20:**
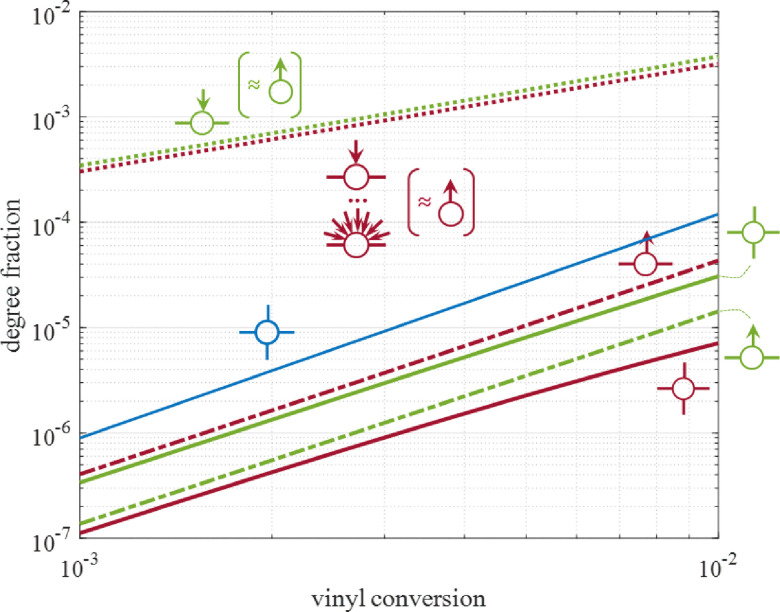
Degree fractions of nodes responsible for linkages in pure HDDA at low vinyl conversion around gelpoint (Fig. 19 shows these over the whole conversion range): blue – without cyclization, green – maximum cycle size 2, red – maximum cycle size 8, high cyclization rate. Normal 4-functional linkages 
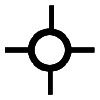
 are suppressed by cyclization and more strongly for larger cycle size. Levels of nodes with ingoing edges (cyclization attacked monomer units) and two undirected edges (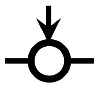 – degree [1, 0, 2] – and 
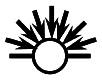
 – degree [7, 0, 2]) – are orders of magnitude higher than both 
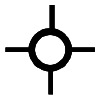
 and nodes with outgoing edges and two undirected edges 
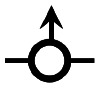
. Levels of ingoing edges match with outgoing edges without undirected edges 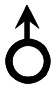, degree [0, 1, 0]. Ingoing edges can only be connected to outgoing edges, but without this distinction connections between two or more attacked units would become abundant.

It has been noticed before that the connectivity structure of the polymer molecules explicitly generated by MC are representative for RG as they are based on the same degree distribution. Storing the connectivity structure of the MC-generated molecule during its generation process in an adjacency matrix allows to use graph theoretical tools and also to visualize the networks. Fig. 13 shows the poly-HDDA networks representative for the RG-model. They may directly be compared to the MD-networks depicted in Fig. 2. What is shown are fragments of the infinite network of about the same size as the MD-networks. They make part of infinite networks and the nodes where the fragments are connected to the further network are marked red.

At left hand side one observes a network of around 400 nodes at the gelpoint, vinyl conversion 0.004. By comparing the MD and RG/MC networks, we see that in both smaller cycles, up to 20, are present in both. Hence, again it may be concluded that introducing a realistic number of cycles into the RG and MC models was successful. Middle and right plots of Fig. 13 recorded at higher conversion well into the gel regime exhibit around 160 cycles up to the maximum size of 20. In green are around 110 cycles that form a tree of adjacent cycles that are connected by at least one edge, as is clarified by the plot at right hand side. Note that according to the underlying model assumption cycles cannot possess nodes in common, hence the closest distance between any two cycles is one edge. A minority of cycles, drawn in blue, is not connected.

Clear differences between the MD and RG graphs are the absence of partly overlapping cycle complexes and large cycles (>100 units) in RG and MC. The first is due to the aforementioned assumption in RG. The occurrence of cycle complexes seems chemically plausible and the RG model must be modified to accommodate these as well. The absence of large cycles would confirm the essentially tree-like character of the network predicted. Also, the (smaller) cycles are connected in a tree-like fashion, both in MD and RG/MC, when regarded on a smaller scale. Note that the large cycles in MD are clearly in contrast with a tree-structure. However, one observes that these cycles happen at the scale of the MD simulation box. To decide, whether large cycles are an artefact, the scaling of such cycles with the size of the simulation box has to be investigated.

## 4 Conclusion

In this work, we have taken a major further step in macroscopic deterministic (random graph) and stochastic (Monte Carlo simulation) modeling of 3D acrylate polymerization by explicitly accounting for cyclization. A new graph-theoretical approach was adopted to enforce cycle representation in the network as clusters bound by directed edges. The distinction between the units in a cycle cluster turned out to be crucial.

First, we successfully used an inverted kinetic Monte Carlo approach proposed by ref. 29 to obtain cyclization rates as a function of cycle size and monomer conversion from detailed molecular dynamics simulations. The order of magnitude of the total cyclization rate competes with the propagation rate, which shows the importance of including it in our model. Then we extended the ARNG methodology generating the population balance equations to account for the new cycle representation. Likewise, we extended the RG model and constructed a new MC sampling model that both generate polymer properties using the degree distribution as input.

Solving the PB equations generated time profiles of the species concentrations and degree distribution, which show the competition between cyclization and propagation reactions. A two-dimensional size/cycle distribution was obtained from the RG-model revealing the many cycles present in the network molecules. Perfect agreement was observed on size distributions, gelpoint and gel fraction results from RG model and MC simulations.

Concerning the gelpoint a small delay was seen to be caused by cyclization. A sensitivity study showed some influence of the maximum cycle size on the delay. Interestingly, this could be correlated to the partial replacement of 4- and 6-functional linkages by combinations of cycle nodes with directed edges also effectively forming higher-functional linkage systems. The importance of distinguishing nodes within cycle clusters was also clearly assessed.

The macroscopic RG and MC models have proved to quantitatively correctly reproduce the presence of cycles of various sizes in the polymer network. Explicit network graphs that we could generate from the MC simulations allowed to compare RG/MC to results from the MD simulations. Around gelpoint few loose pending cycles are present, but in more developed networks in gel regime cycle clusters are predicted. Although in MC-generated networks the minimum distance between two adjacent cycles is one edge, they reveal the existence of large trees of such adjacent connected cycles. However, the clusters in MD also contained complex overlapping smaller cycles. Partly due to simplifying assumptions made in RG/MC, such complexes were not reproduced there. In future RG/MC model development these will be relaxed by allowing cycles to share nodes and edges. Another difference observed was the presence in the MD structures of larger cycles (more than 100 monomer units), which contradicts a true tree-like structure. RG essentially does predict trees, even the connected cycles have this structure. Large cycles occur on the scale of the box size, so the relation between box and cycle size should be investigated further.

It should finally be noted that we have taken a major step forward in predictability of the RG model with respect to network structure. However, we still had to rely on estimates of the strongly decreasing reaction rates from MD simulations. The ultimate goal of RG modeling, to become predictive of the decreasing rates through the changing structure during polymerization it is now able to describe, still remains for the future.

## Conflicts of interest

There are no conflicts to declare.

## Figures and Tables

**Fig. 16:**
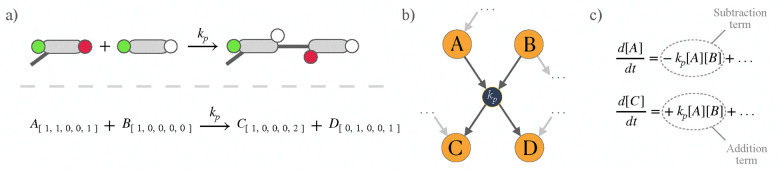
The transformation from chemical knowledge into reaction network (RN) and PB equations. (a) A propagation reaction between two available monomer species, translated in a notation that focuses on the reactive parts. (b) The same reaction as in (a), but translated into RN, where species nodes (orange) connect *via* reaction nodes (blue). (c) Using the RN in (b), PBEs for all unique monomer species are constructed that contain subtraction and contribution terms for all reactions they partake in. Red dots: radical; green dots: vinyl group; white dots: reacted vinyl.

## Data Availability

The molecular dynamics data (time series of adjacency matrices) and 50–100 MATLAB routines containing various parts of the ARNG- and RG-models are available upon request.

## Appendices

### Appendices

#### A Appendix

##### A.1 Kinetics of photopolymerization of multifunctional acrylates

The kinetic scheme for the case of HDDA polymerization is shown in Fig. 14. In acrylate photopolymerization of dissociation is induced by laser light, photo initiation (1). In the case of multifunctional acrylates such as HDDA and TMPTA, radicals propagate through the system by reactions with unbound monomers, extending the polymer chain *via* a reaction between the radical and one of the free monomer's vinyl groups, while the other non-reacting vinyl(s) become free-pending double bonds. Reactions between radicals and free pending double bonds create linkages (3). Finally, two termination processes eliminate radicals from the system by either disproportionation (4), where no link is formed, or recombination (5), which produces a linkage. The radical propagation process is the main source of branch formation and, ultimately, the creation of a polymer network.

The mobility of a monomer with radical is limited to its close neighborhood and is very likely to react with a free pending double bond on its own chain.^26^ These reactions produce a linkage within the chain, forming a cycle, and are therefore called cyclization reactions, see Fig. 14 (6). Note that acrylates also feature a backbiting reaction, which is the transfer of a radical on a primary C-atom to a C-atom back in the chain. In the present study (as in ref. 4) we do not take this backbiting into account.

Although cyclization reactions lead to identical chemical bonds as radical-vinyl propagation reactions, they differ from a few other perspectives. Firstly, regular propagation is second-order, where the rate is proportional to the product of radical and vinyl group concentrations. In contrast, cyclization is a first-order reaction and the rate is only proportional to the radical concentration, since cycles form between a radical at the end of a chain and vinyl groups along the same chain (see also ref. 39–41). Cycles of varying sizes can be created, depending on the intrachain distance between the radical and the pending double bond on its own chain. This size has been observed to vary from mini-cycles within a single monomer to larger structures up to 14 monomer species^26^ for the case of HDDA. It was also observed in the MD simulation that cyclization may happen by termination by two radicals sitting on the same chain, be it less frequently than propagation cyclization.

Arguably, the impact of cycles on network topology will depend on their size – the number of monomer units in the cycle. Monocycle formation, or the cycle that is formed by the reaction of a radical and a vinyl group on the same monomer, and subsequent ‘regular’ propagation results in linear polymer chains without free pending double bonds and effectively prevents branching. Larger cycles do not involve both functional groups on a monomer unit, leaving vinyl groups intact that can participate in other linking reactions. In this paper, we address the formation of cycles of varying size to investigate the effect on network formation, thereby using the kinetic rates of cyclization from MD data.

##### A.2 Construction of reaction network in ARNG algorithm

Fig. 16 shows the translation of a chemical reaction – a propagation reaction – into part of the reaction network and finally to PBE terms. Species nodes in the reaction network (orange) do not directly connect with each other. Rather, when they serve as reactants in a reaction, out-going directed edges connect them to the relevant reaction node (blue), from which out-going edges connect to the relevant product species nodes.

The reaction network graph in itself is mathematically represented by an adjacency matrix, *A*, which has size (*n*_*s*_ + *n*_*r*_) × (*n*_*s*_ + *n*_*r*_), for *n*_*s*_ species and *n*_*r*_ reactions, from which the PBEs are derived. Following the method by ref. 45 as applied and described before in ref. 5 and 35, PBEs contain addition and subtraction terms based on the reactant concentration(s) and the rate coefficients – see Fig. 16c. Solving the PBEs yields the concentration profiles over time of all *n*_*s*_ species, *c*(*n*_*j*_, *t*), *j* = 1:*n*_*s*_.

##### A.3 Reaction rules

The rules of all reactions except cyclization are presented in Table 4 using the vector notation of the species. These rules correspond to all the reactions in Fig. 14 and only change the number of vinyls, radicals and undirected (undirected) links of the species. The construction of the reaction network in general starts with just two initial species: the initiator I_2_ and unreacted monomer with two vinyl groups *n* = [2, 0, 0, 0, 0], see Fig. 15. By applying the reaction rules in Table 4 to the species present, new species are generated that can react further.

**Table 4:** Reaction rules for free radical polymerization of multiacrylates for all reactions except cyclization. The reaction types are listed with the requirements for the reactant(s) for them to occur, which are shown in the reactant column(s). The rightmost column with reaction equations describes the change of state(s) of the reactant units. The representation used for species types is according to Fig. 15. Note that these reactions do not change the in-coming and out-going indices of the vectors

#	Reaction	Order	*R*1	*R*2	Rate coeff.	Reaction equation
1	Initiator dissociation	1	I_2_		*k*_*d*_	I_2_ → 2I˙
2	Initiation	2	*v* > 0	I˙	*k*_*i*_	[*v*, *r*, *i*, *o*, *u*]_*R*_1__ + I˙ → [*v* − 1, *r* − 1, *i*, *o*, *u*]_*P*_1__
3	Propagation	2	*v* > 0	*r* > 0	*k*_p_	[*v*, *r*, *i*, *o*, *u*]_*R*_1__ + [*v*, *r*, *i*, *o*, *u*]_*R*_2__ → [ *v* − 1, *r* + 1, *i*, *o*, *u* + 1]_*P*_1__ + [*v*, *r* − 1, *i*, *o*, *u* + 1]_*P*_2__
4	Termination dispropor-tionation	2	*r* > 0	*r* > 0	*k*_td_	[*v*, *r*, *i*, *o*, *u*]_*R*_1__ + [*v*, *r*, *i*, *o*, *u*]_*R*_2__ → [*v*, *r* − 1, *i*, *o*, *u*]_*P*_1__ + [*v*, *r* − 1, *i*, *o*, *u*]_*P*_2__
5	Termination recombination	2	*r* > 0	*r* > 0	*k*_tc_	[*v*, *r*, *i*, *o*, *u*]_*R*_1__ + [*v*, *r*, *i*, *o*, *u*]_*R*_2__ → [*v*, *r* − 1, *i*, *o*, *u* + 1]_*P*_1__ + [*v*, *r* − 1, *i*, *o*, *u* + 1]_*P*_2__

The cyclization reaction rules are listed in Table 5 grouped by the reactants, to which they apply. In grey the complete reaction equations are displayed for a triacrylate system. For cycles of size 2, two adjacent monomer units react, one containing a radical and the other a vinyl, in addition to at least one undirected edge. When the reaction takes place, the vinyl disappears from the vinyl-containing species and the radical moves to the other monomer. At the same time, out-going edges replace one undirected edge of both species. For cycles ≥3, the same reacting radical and vinyl monomers are used, but their placement is different in the polymer chain, while there is also at least one intermediate monomer unit. As one possible instance of the table, as reactants we might take the radical at the 4th row, [1, 1, 0, 0, 1] reacting with the 6th row in the vinyl column, [1, 1, 0, 0, 2] reacting to a cycle of size 6, leading to products [0, 1, 0, 1, 0] and [0, 1, 5, 0, 1] This involves four intermediate units, which should be species of the third column, for instance two times [1, 0, 0, 0, 3] plus [0, 0, 0, 6] and [2, 0, 0, 0, 2]. All combinations of monomers with radicals and monomers with vinyls are possible in cyclization reactions. Since the only requirement of that monomer is the presence of at least two undirected edges, the inclusion of cyclizations of larger size leads to a great number of reactions, since all combinations of the *v*, *r*, *u* species are possible.

**Table 5:** Reaction rules for cyclizations ≥2 in a triacrylate system. On the first line, in gray, the reactant vectors undergoing cyclization are shown. Below, for the reacting radicals, vinyls and changing intermediate units the [*v*, *r*, *u*] parts of all configurations that can participate in the cyclizations are shown. Only cycles of size 3 and larger involve changes of intermediate units. Any combination of radical, vinyl and non-reacting unit configurations is possible

Cycle size *n*_*c*_	Reacting radical			Reacting vinyl			Intermediate unit		
	*v*	*r*	*u*	*v*	*r*	*u*	*v*	*r*	*u*
2 & ≥ 3^a^	[*v*, *r*, 0, 0, *u*] → [*v*, *r* − 1, 0, 1, *u* − 1]			[*v*, *r*, 0, 0, *u*] → [*v* − 1, *r* + 1, *n*_*c*_ − 1, 0, *u* − 1]			[*v*, *r*, 0, 0, *u*] → [*v*, *r*, 0, 1, *u* − 2]		
	0	1	1	1	0	1	0	0	2–6
	0	2	1	1	0	2	0	1	2–5
	0	3	1	1	0	3	0	2	2–4
	1	1	1	1	0	4	0	2	2, 3
	1	2	1	1	1	1	1	0	2–4
	2	1	1	1	1	2	1	1	2, 3
				1	1	3	1	2	2
				1	2	1	2	0	2
				1	2	2			
				2	0	1			
				2	1	1			

a Only cycles of size 3 and larger require an additional non-reactive unit that links the reacting radical and the vinyl containing monomers, given in the third column.

##### A.4 Rate equation for cyclization

Cyclization reactions are first-order in the head radical attacking a vinyl unit on the same chain. However, complicating issues arise concerning the availability of the vinyl units and the intermediate units. All species in a cyclization reaction can be either saturated or reactive, and is either the species with a reacting radical, the species with a reacting vinyl or an intermediate species. From the ARNG, a complete list of species is known. For easy notation, we define *R* the set of indices of species that can have possibly reacting vinyls, *I* the set of indices of species that can be intermediate species, *H* the set of indices of species that can have head radicals, and *S* the set of indices of species that are unreactive (saturated). Note that *R*∩*S* = ∅ and *H*∩*I* = ∅ as a monomer cannot be reactive and unreactive or intermediate and have a head radical. All other combinations are possible in principle. We will be unpacking the rate formula for a cyclization reaction for species *s*∈*H* for a cycle of size *l* ≥ 3, which is first order. The rate is given by

8

It is a multiplication of 5 terms. *k*_*l*_, the size (*l*)-dependent rate of cyclization together with *c*_*s*_, the concentration of species *s*, are the usual first order terms. There are correction terms however, as not all species have a possibly reacting vinyl and can hence be the attacked node in the cyclization reaction. This fraction of possible reactive species compared to the total amount of species is given by . For a cycle of size *l*, there are *l* − 2 intermediate species. Each individual species has fraction *f*_*i*_ of the total possible intermediate species. As the *l* − 2 linking species can be any combination of species from *I*, we use the multinomial distribution to determine the probability of finding a specific set [*x*_1_,…*x*_*l*−2_], *x*_*i*_ ∈ *I*. This probability is given by the fourth and fifth term in eqn (8). An example follows with the following cyclization reaction creating a 4-cycle:

9

with intermediate species *i*_1_, *i*_2_ transforming into  in the ARNG notation. Suppose that ARNG yields |*I*| = 6 = {*n*_1_,…,*n*_6_} as the number of possible intermediate units. Then we inquire after a specific reaction, where the aforementioned radical and vinyl species are reacting simultaneously with 2 out of the 6 possible units: lets say belonging to species indices *n*_3_ and *n*_5_. Eqn (8) and *l* − 2 = 4 − 2 = 2 then reads:

10

How to deal with these to obtain the proper rate equations will be explained using the following reaction as an example:

11 [0, 3, 0, 0, 1] + [1, 0, 0, 0, 4] + [1, 1, 0, 0, 3] → [0, 2, 0, 1, 0] + [0, 1, 3, 0, 3] + [1, 1, 0, 1, 1]

The reaction above describes a cyclization of size 3 that reacts with a rate of *k*_*n*_3__. It should be realized that only part of the head radicals can perform this cyclization as non-reactive units (*e.g.* 3- or 4-functional linkages) may occupy the position of 3 units away from the head. Hence, the total formation rate of cycles of size 3 originating from radical species [0, 3, 0, 0, 1] equals to  where  is the sum of the concentrations of unreactive species and  is the sum of units with possibly reacting vinyls. Furthermore, the reaction equation above presumes that the vinyl unit on [1, 0, 0, 0, 4] is reacting, but vinyls on other units located at the position in question may react as well (middle column Table 5 of the main text). This brings the total of [0, 3, 0, 0, 1] forming cycles of size 3 to: .

Finally, the reaction equation above presumes the intermediate unit [1, 1, 0, 0, 3] to participate in this specific cyclization reaction, but other intermediate unit types like [1, 1, 0, 0, 2] may participate as well (rigthmost column of Table 5 of the main text). Hence, to obtain the rate involving this specific intermediate unit requires multiplying with the fraction  where the numerator counts over all possible intermediate units, *k* = 1, …, *N*_*i*_. As in the example of a 3-cycle only one intermediate unit is involved (*n*_int_ = 1) the multiplication is a simple fraction. However, more intermediate units require multiplication by the probability of finding a specific set (with number of elements *n*_int_) [*x*_1_, *x*_2_, …, *x*_*N*_*i*__] out of all *N*_*i*_ possible intermediate units. This is given by a multinomial distribution, which is part between square brackets in the general formula for the rate of size *c*_*s*_ cyclization:

12

Here, *f*_*n*_1__ to  denote the fractions of possible intermediate units: *etc.* For instance, consider a similar situation as before in Equation (9) with radical species [0, 3, 0, 0, 1] reacting with vinyl species [1, 0, 0, 0, 4], but now creating a larger cycle: size *c*_*s*_ = 4, corresponding to *n*_int_ = 2 intermediate units. Suppose that ARNG yields *N*_*i*_ = 6 as the number of possible intermediate units. Then we inquire after a specific reaction, where the aforementioned radical and vinyl species are reacting simultaneously with 2 out of the *N* = 6 possible units: *n*_3_ and *n*_5_. Eqn (12) with *N* = 6 and *n*_int_ = 2 then reads:

13

##### A.5 MD extracted moving rate averages of all cyclization reactions

Table 6 contains the polynomial coefficients for the number of cyclization events per time unit, *z*_*c*_, in the reaction volume simulated by MD. This underlies the cyclization rate coefficients as shown in Fig. 6 and 7 in the main text. Fig. 17 shows the raw cyclization events data *z*_*c*_ (left), the much more frequent propagation events *z*_p_ (right, in yellow) and the less frequent termination events *z*_t_ (right, in red).

**Table 6:** Polynomial coefficients per cycle size of number of cyclization events as a function of vinyl conversion, *χ*: *z*_*c*_ = *P*_0_*χ*^3^ + *P*_1_*χ*^2^ + *P*_2_*χ* + *P*_3_

Cycle size	*P*_0_	*P*_1_	*P*_2_	*P*_3_
1	17.099	−11.978	−11.491	8.1967
2	6.6793	−1.3047	−12.929	7.9560
3	31.393	−45.681	9.6603	5.7109
4	76.645	−119.40	41.037	4.6423
5	34.745	−67.610	29.627	2.1550
6	91.081	−150.71	62.826	−0.0457
7	68.274	−115.56	47.596	1.4049
8	44.955	−84.454	38.974	0.28563
9	19.099	−53.069	31.583	−0.66653
10	53.385	−91.244	39.164	−0.0586
11	−3.0284	−7.5878	8.3560	−0.21852
12	27.503	−49.246	21.951	−0.10087
13	25.561	−44.278	19.882	−0.60265
14	7.2757	−18.750	10.951	−0.48760
15	42.244	−63.509	23.804	−0.00327
16	14.545	−22.855	9.3072	−0.27619
17	14.903	−24.531	10.525	−0.34553
18	13.713	−21.112	8.0926	0.019150
19	19.809	−29.901	11.216	0.013027
20	14.972	−23.509	9.5524	−0.27550
21	6.6388	−10.273	3.9978	−0.0183
22	5.1002	−7.8937	3.2064	−0.12864
23	5.4826	−9.4584	3.9960	0.038195
24	20.798	−29.907	10.536	0.058091
27	6.4361	−10.031	4.0770	−0.14217
28	17.730	−25.217	8.7946	0.024216
29	3.1815	−4.7517	1.8310	−0.0541
30	6.3629	−9.5034	3.6621	−0.10822
32	6.1988	−9.2193	3.5152	−0.0883
40	3.6487	−5.2724	1.9120	−0.02395
43	3.6487	−5.2724	1.9120	−0.02395
44	3.6256	−4.9603	1.5698	0.063114
46	7.2512	−9.9207	3.1396	0.12623
54	2.0608	−2.2774	0.18540	0.25579

Concerning rate coefficients for propagation and termination we have adopted the values for termination from ref. 29 and the values for propagation from the same source as well, but the latter with a correction for cyclization events. In ref. 29 all reaction events consuming vinyl groups, *z*_*v*_, were considered propagation events, so *z*_p_ = *z*_*v*_, which was then used in the 2nd-order inverted kMC-equation:^29^

14

(*x*_*v*_(*χ*) and *x*_*r*_(*χ*)) being the numbers of vinyls and radicals in the MD-simulation at vinyl conversion *χ*, *V* and *N*_A_ the reaction volume and (Avogadro's number, respectively). However, cyclization events *z*_*c*_ in eqn (2) also consume vinyl groups, which implies that a smaller number of the reacting vinyl groups than *z*_*v*_ should be attributed to propagation: *z*_p_ = *z*_*v*_ − *z*_*c*_. In the present PB model we use eqn (14) with the smaller value of *z*_p_ to estimate the corrected value of the propagation rate coefficient. Finally it is assumed that the propagation, termination and cyclization rate coefficients for the mono- and tri-acrylates, NBA and TMPTA, are equal to those for HDDA.

The RHS of Fig. 17 will finally be used to illustrate the principle of the inverted kinetic Monte Carlo procedure to estimate the rate coefficients from counts of reaction events and reactant molecules. This plot of raw reaction data has a resolution of 100 points covering total reaction time of 6.6 × 10^−9^, so reaction time interval is *τ* = 6.6 × 10^−11^. At a time interval at one-third of the reaction time, 2.2 × 10^−9^, the figure shows that *z*_p_ = 18 propagation events happen, while just one termination event occurs, *z*_t_ = 0.5. (This is counted as 0.5, since the event is detected as the disappearance of just one radical, while each full termination step consumes two.) By the propagation reaction the number of vinyls, *x*_*v*_ (originally 3900), decreases from 1080 to 1062, while the number of radicals *x*_*r*_ reduces from 74 to 73. With a reaction volume *V* = 7.5 × 10^−25^ m^3^, activation energies *E*_a,p_ = 31.02 × 10^3^ J mol^−1^, *E*_a,t_ = 8.67 × 10^3^ J mol^−1^ and temperature 300 K we apply eqn (14) to find, at a vinyl conversion *χ* = 0.75, values *k*_p_ = 6300 L m^−1^ s^−1^ and *k*_t_ = 1.36 × 10^6^ L m^−1^ s^−1^ (see also Fig. 6).

##### A.6 linkage, cycle and degree distribution profiles

In Fig. 18 indicated in red and yellow are concentrations of 4- and 6-functional linkages, respectively. Linkage concentrations reach levels comparable to the initial acrylate concentrations. Comparing the 4- () and 6-functional () linkage curves in absence of cyclization to those for small cycles (size 1 and 2) shows that they are (almost) equal. Remarkably, assuming larger cycles (up to size 5) 4- and 6-functional linkages attain concentrations much lower – by several orders of magnitude. This is caused by the consumption of these linked units as intermediate units by cyclizations larger than two. These units are thereby changed into cycle nodes with outgoing edges  for HDDA and  for TMPTA. This is shown in the bottom plot, where, for HDDA, one observes the larger cycles producing  (darker green) at higher level than small cycles (lighter green). A similar trend is seen for the  from TMPTA by comparing the darker to the lighter blue curves.

Fig. 19 displays the degree distribution *versus* vinyl conversion for the case of HDDA polymerization with cycle sizes 1–8 forming according to MD-rates (total rate equal to total rate of all cycles observed in MD). In the figure in blue are the normal nodes of total degrees 1, 2, 3, 4. Those of degree and 2 (linear chain segments) are most abundant at low conversion but at high conversion levels of 4-and 2-functional nodes are equal, indicative of a highly dense network. In yellow cycle nodes with an outgoing edge, at higher level with one normal edge (fat) than with two normal edges (thin). Similar trends are observed for the cycle nodes with two normal edges and from one to 7 incoming edges: between  and  (red) and those with one normal edge (green). Since cyclization rate decreases with size, fewer nodes with a larger number of incoming edges are present. In blue with increasing numbers of normal edges, degrees *k* = [*i*, *o*, *u*] = [0, 0, 1], [0, 0, 2], [0, 0, 3], [0, 0, 4]. In yellow shown are cycle nodes with an outgoing edge, at higher level with two normal edges (fat, degree [0, 1, 2]) than with one normal edge (thin, degree [0, 1, 1]). Note that at high conversion the fraction of [0, 1, 2] nodes – with total linking functionality 4 – considerably exceeds the level of the non-cycle nodes [0, 0, 4]. Hence, one observes that the reduced connectivity by fewer [0, 0, 4] nodes is more than compensated by the 4-functional cycle nodes [0, 1, 2]. Cycle nodes with varying numbers of incoming edges between  (degree [1, 0, 2]) and  (degree [7, 0, 2]) and two normal edges in red and those with one normal edge in green (degrees [1, 0, 1] and [7, 0, 1]) are observed at lower levels. Note that the total numbers of incoming edges by definition must be equal to the total of outgoing edges.

##### A.7 Gelpoint delay and degree distribution

Gelation is primarily caused by 4-functional linkages , which are reduced by cyclization, as shown in Fig. 20. However, combinations of nodes in a cycle may effectively lead to high-functional linkages. At the low conversion around gelpoint the levels of nodes with ingoing edges and two undirected edges ( – degree [1, 0, 2] – and  – degree [7, 0, 2]) – are found to be orders of magnitude higher than both  and nodes with outgoing edges and two undirected edges . It should be realized that the combination of such edges to nodes with outgoing edges and two undirected edges () effectively would lead to 4-functional linkages as well. However, in this conversion region the levels of such nodes is orders of magnitudes lower. This indeed partly compensates the reduction of , but to a limited extent. We see that the high concentration of  and  predominantly will combine with the equally high levels of nodes with outgoing edges without undirected edges,  – degree [0, 1, 0], which do not lead to 4-functional node combinations. It is clear that in absence of the distinction between in- and outgoing cycle nodes the RG-algorithm would be allowed to connect high levels of nodes both with ingoing edges, which would lead to high concentrations of 4-functional nodes. In a variant of our RG-model without the distinction between attacked and non-attacked units we observed the gelpoint indeed to be situated at extremely low vinyl conversion – as low as 10^−6^.
